# Supplementary figures and images for: Octopamine signaling regulates the intracellular pattern of the presynaptic active zone scaffold within Drosophila mushroom body neurons
Source: PLoS Biol. 2025 Oct 23;23(10):e3003449. doi: 10.1371/journal.pbio.3003449 (PMC12626334; doi:10.1371/journal.pbio.3003449)

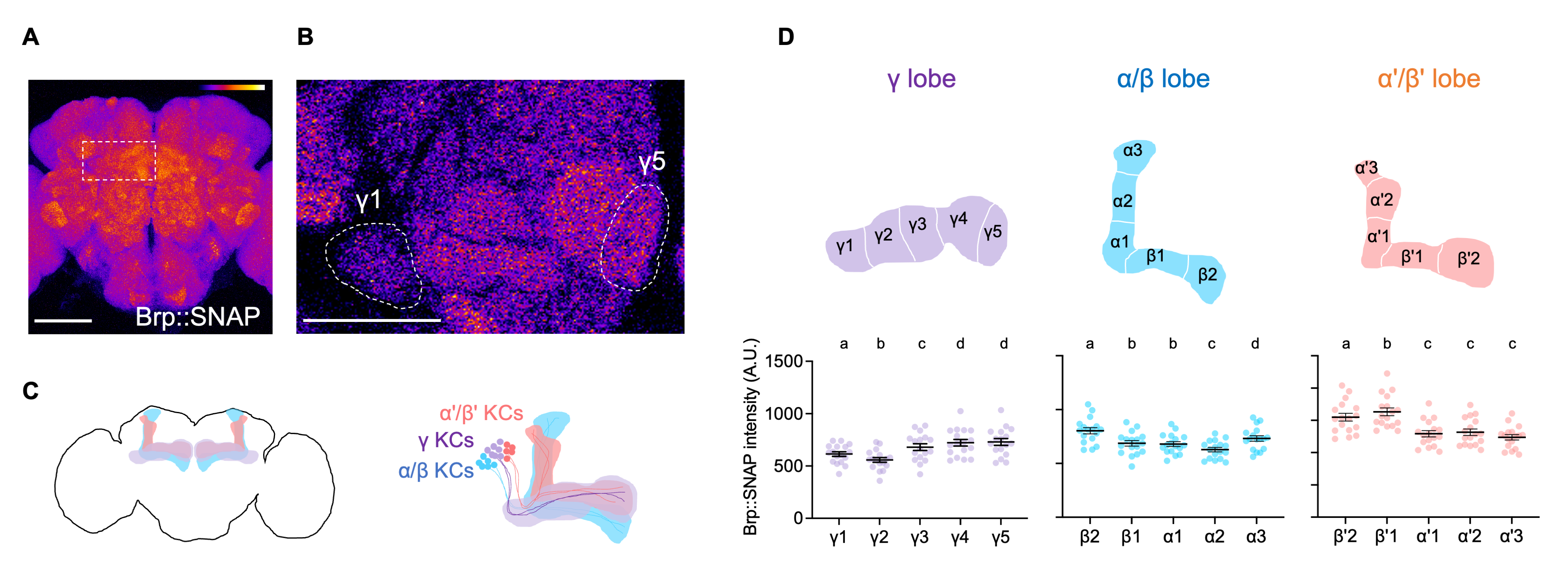

Supplement: S1 Fig — (A) SNAP chemical tagging labeled endogenous Brp in the brain. Scale bar, 100 μm. The dashed line area indicates the zoomed-in area shown in (B). (B) Intensity difference of Brp::SNAP between γ1 and γ5 compartment on the same imaging plane. Scale bar, 50 μm. (C) Schematic of the MB. The MB comprises three lobes based on the projection patterns of three KC subtypes: γ KCs, α′/β′ KCs and α/β KCs. (D) Brp::SNAP intensity difference across compartments. Schematic drawings above indicate compartments of each lobe. Error bars show S.E.M. Significant differences (P < 0.05) are indicated by distinct letters. Repeated measures one-way ANOVA. The data underlying this Figure can be found in S1 Data. (TIFF) [file pbio.3003449.s002.tiff]

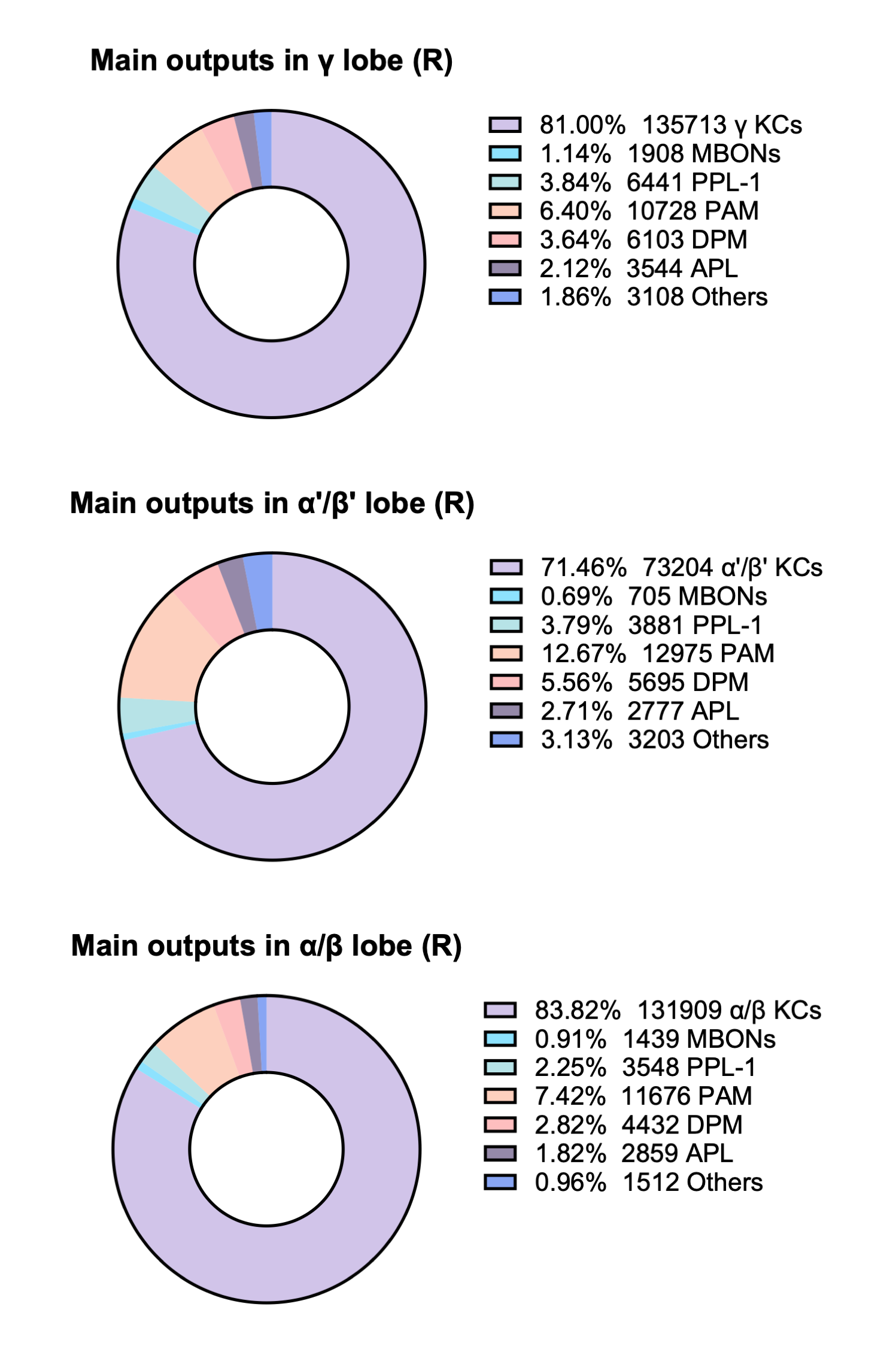

Supplement: S2 Fig — Pie charts showing the composition of pre-synapses in each of the three MB lobes. Data is from the hemibrain online data (not all the cell types are listed). The number of pre-synapse and the percentage are indicated for each cell type, including KCs, MBONs PPL-1 neurons, PAM neurons, dorsal paired medial (DPM) neuron and anterior paired lateral (APL) neuron. (TIFF) [file pbio.3003449.s003.tiff]

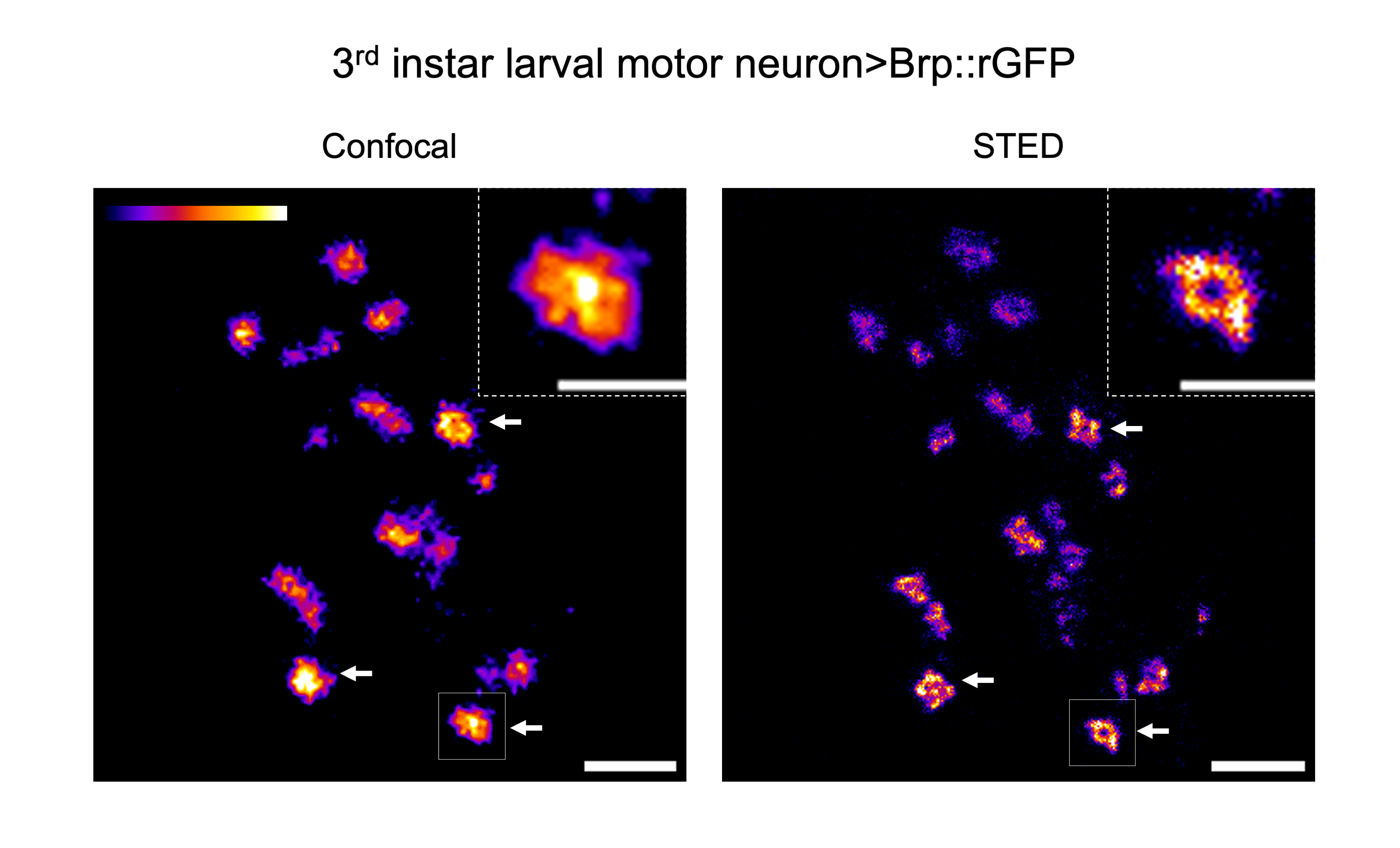

Supplement: S3 Fig — White arrows indicate single AZs that show donut-shape in the STED image. Scale bar, 1 µm in the overviews; 500 nm in the insets. (TIFF) [file pbio.3003449.s004.tiff]

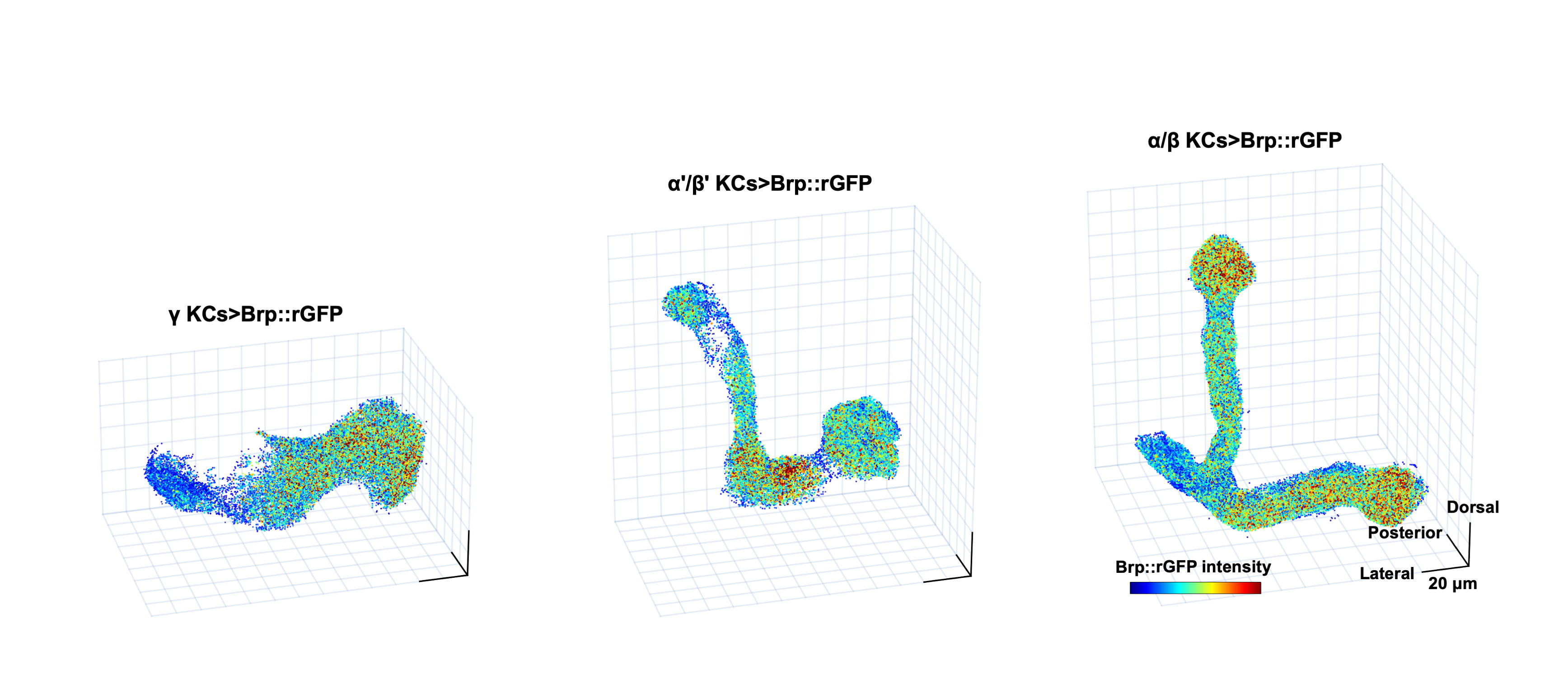

Supplement: S4 Fig — The 3D reconstruction of Brp::rGFP clusters, colored by Brp::rGFP intensity. Brp::rGFP is visualized in γ KCs using MB009B-GAL4, in α/β KCs using MB008B-GAL4 and in α′/β′ KCs using MB0370B-GAL4. Scale bars, 20 μm. (TIFF) [file pbio.3003449.s005.tiff]

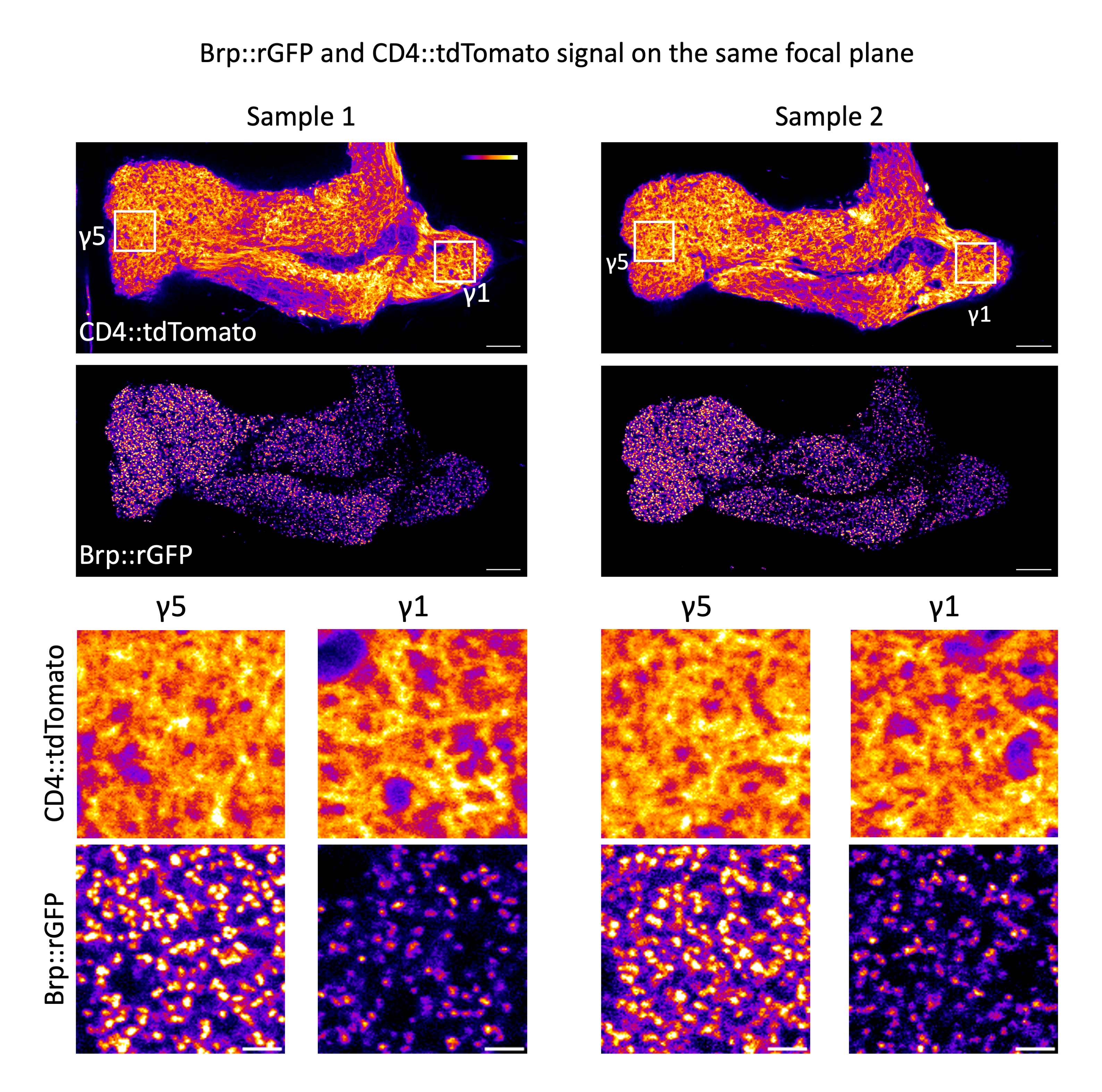

Supplement: S5 Fig — Two independent samples are shown. While tdTomato intensities are similar in both compartments, Brp::rGFP is weaker in γ1, indicating the Brp heterogeneity is not due to imaging depth difference. Upper panels, single slice in the image stack where both γ5 and γ1 are shown. White boxes indicate zoomed-in areas shown in the lower panels. Scale bar, upper panels: 10 µm; lower panels: 2 µm. (TIFF) [file pbio.3003449.s006.tiff]

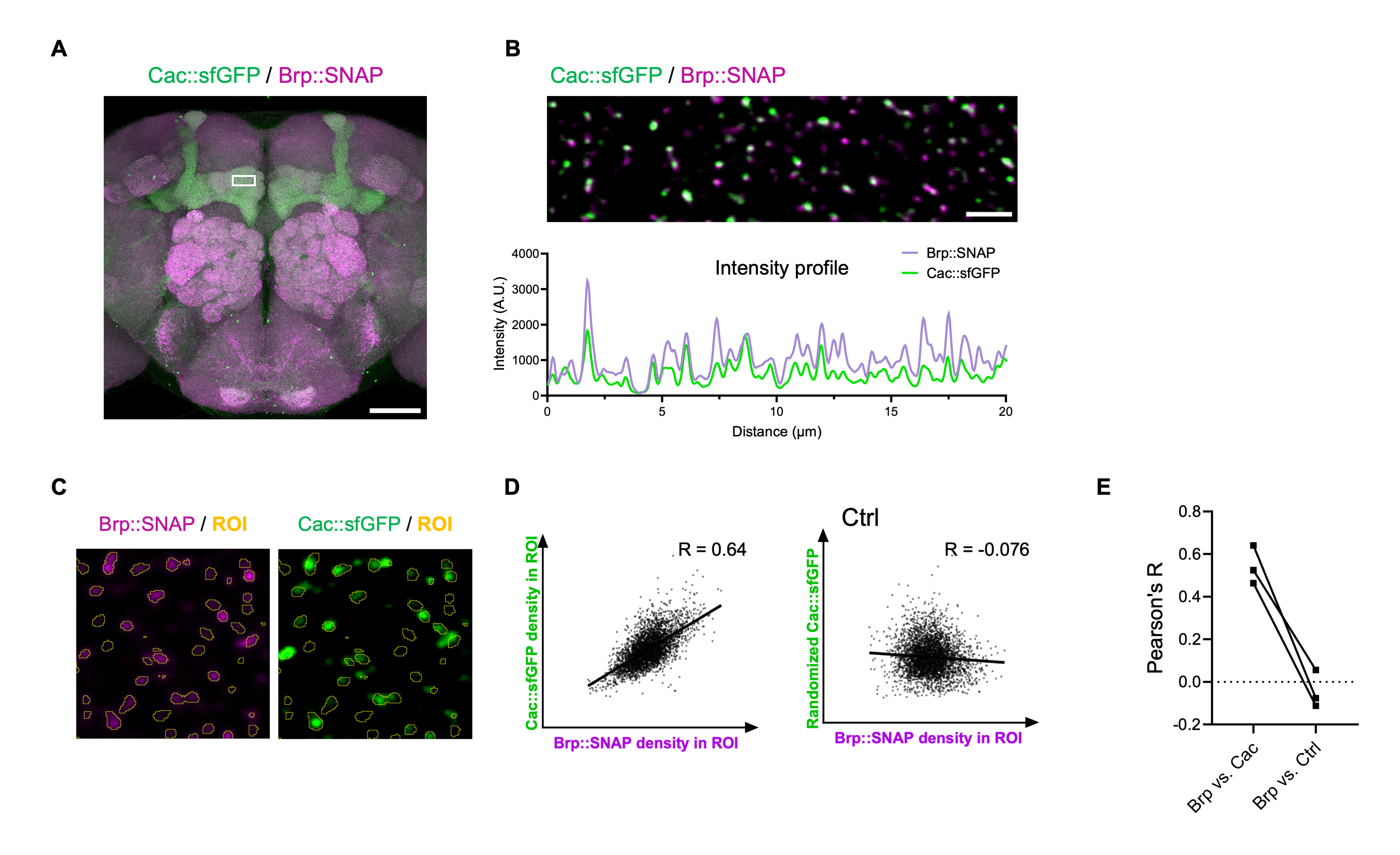

Supplement: S6 Fig — (A) Co-labeling of Cac::sfGFP (green) and Brp::SNAP (magenta) in an adult brain. The write box indicates the zoomed-in areas shown in (B). Scale bar, 50 μm. (B) Co-localization of Brp::SNAP and Cac::sfGFP signals. Signal intensity profiles of both Brp::SNAP (green) and Cac::sfGFP (magenta) in the image were plotted below. Scale bar, 10 μm. (C) Correlation between Brp::SNAP (green) and Cac::sfGFP (magenta) signal intensities. The image is a selected area from γ5. Yellow circles show the 3D ROIs generated by segmenting Brp::SNAP signals. A loose setting was applied to include surrounding pixels. The same ROI set was used to quantify the signal density (total grey value divided by the ROI volume) for both Brp::SNAP and Cac::sfGFP. (D) Scatter plot showing the correlation between Brp::SNAP and Cac::sfGFP signal intensities in an image sample. A 180° rotated Cac::sfGFP image was used as a control (see also S7 Fig). Pearson’s correlation coefficient (R) is shown. (E) Pearson’s correlation coefficient (R) from three individual γ lobes showing the correlation between Brp::SNAP and Cac::sfGFP signal intensities. Data are represented as box plots showing center (median), whiskers (Min. to Max.). Significant differences (P < 0.05) are indicated by distinct letters. Kruskal–Wallis test. The data underlying this Figure can be found in S1 Data. (TIFF) [file pbio.3003449.s007.tiff]

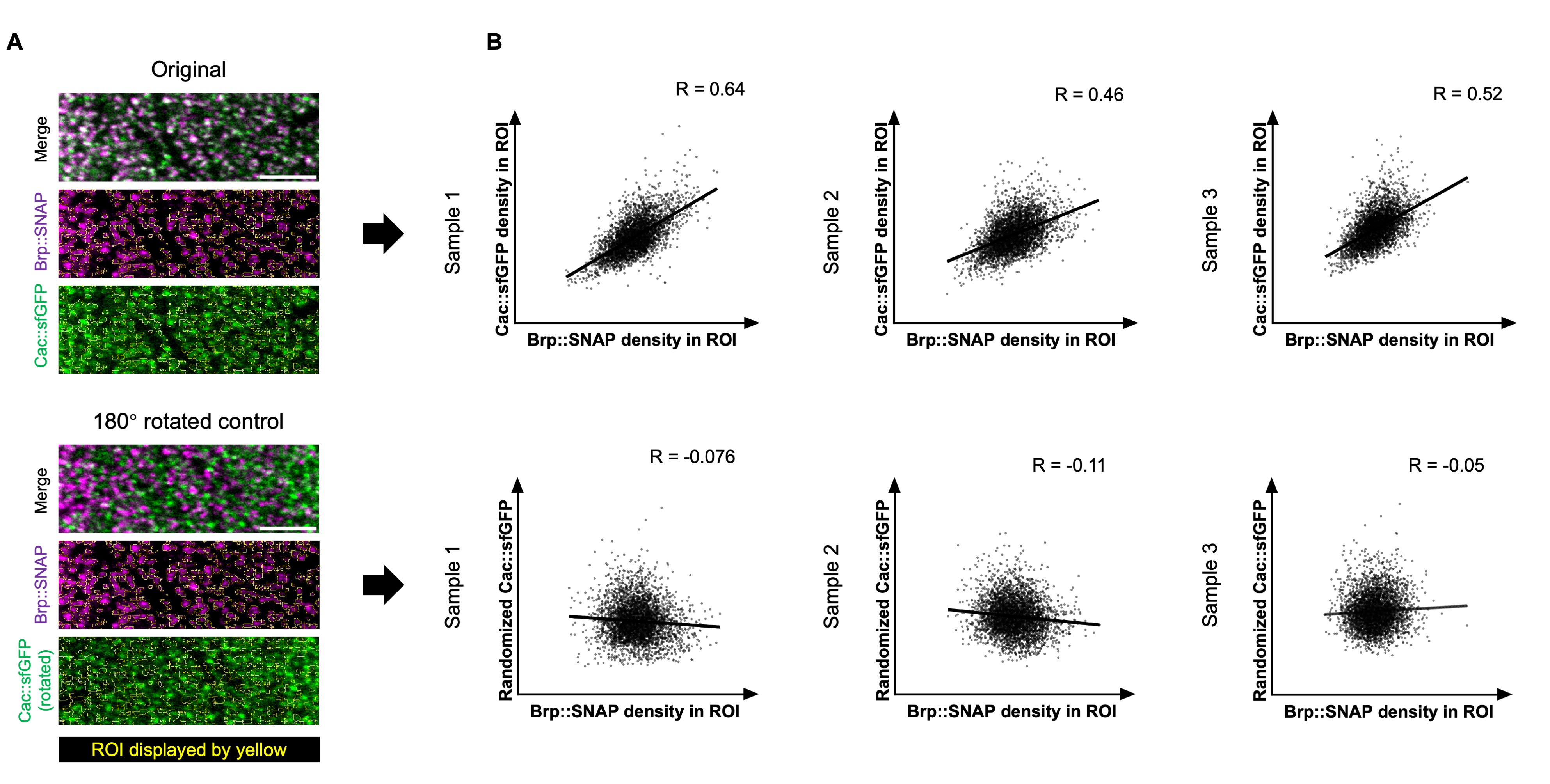

Supplement: S7 Fig — (A) Correlation analysis of Brp::SNAP and Cac::sfGFP signal intensities. ROIs are generated using 3D spot segmentation method without a watershed process. Relatively loose setting was applied on Brp::SNAP images to generate wide ROIs. The same ROI set was used to calculate signal intensities in both Brp::SNAP and Cac::sfGFP channels. The 180° rotated version of Cac::sfGFP image was used as the control. A total of 4,000 ROIs were analyzed, and Pearson’s correlation coefficient (R) values were calculated for each sample. Scale bars, 5 μm. (B) Scatter plots showing the correlation between Brp::SNAP and Cac::sfGFP signal intensities (left) and control (right) in different brain samples. R value is indicated for each sample. (TIFF) [file pbio.3003449.s008.tiff]

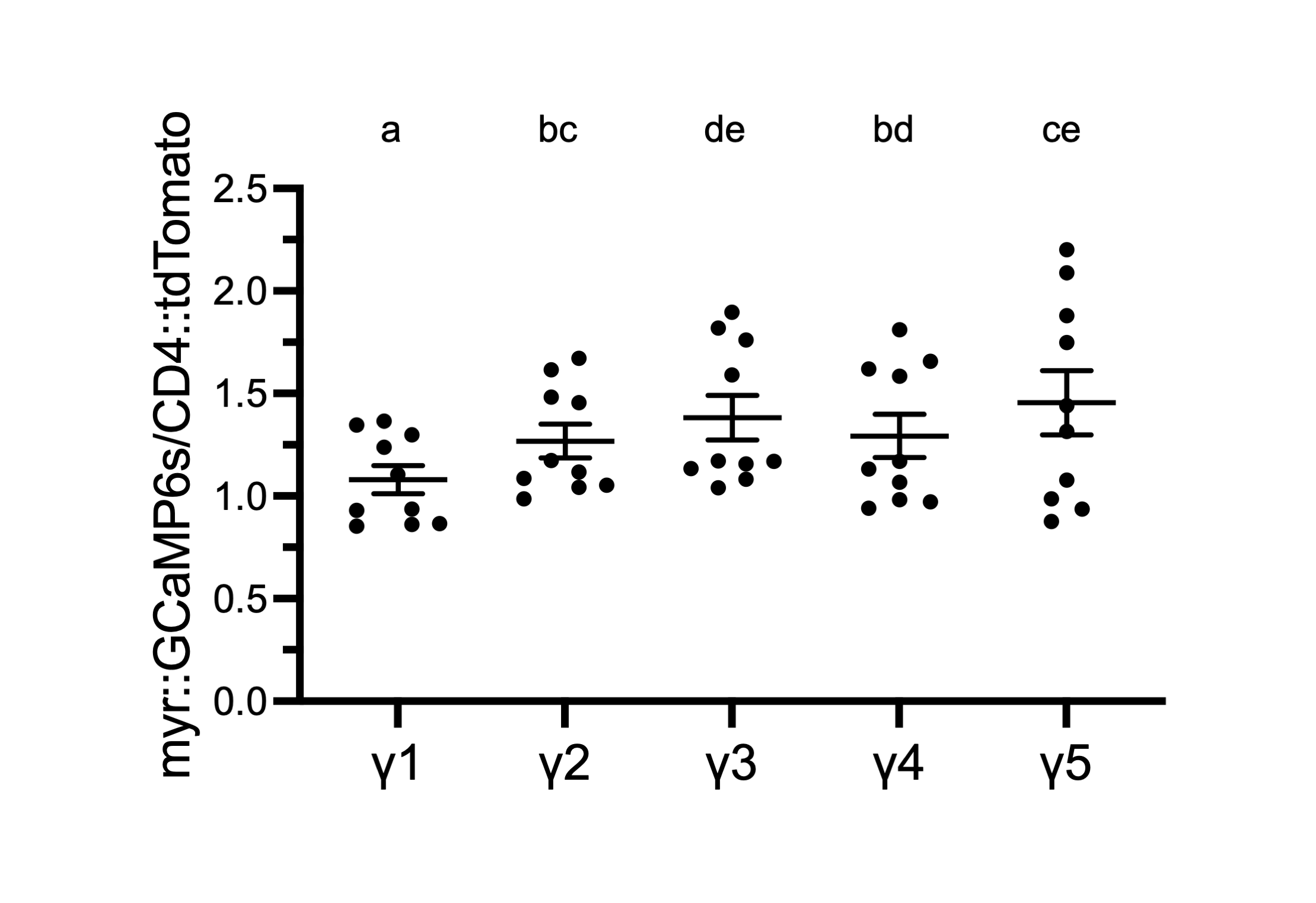

Supplement: S8 Fig — The ratio of myr::GCaMP6s to CD4::tdTomato in γ compartments is shown. Experimental procedures and quantification are comparable to that of Fig 2E. Error bars show S.E.M. Significant differences (P < 0.05) are indicated by distinct letters. Repeated measures one-way ANOVA. The data underlying this Figure can be found in S1 Data. (TIFF) [file pbio.3003449.s009.tiff]

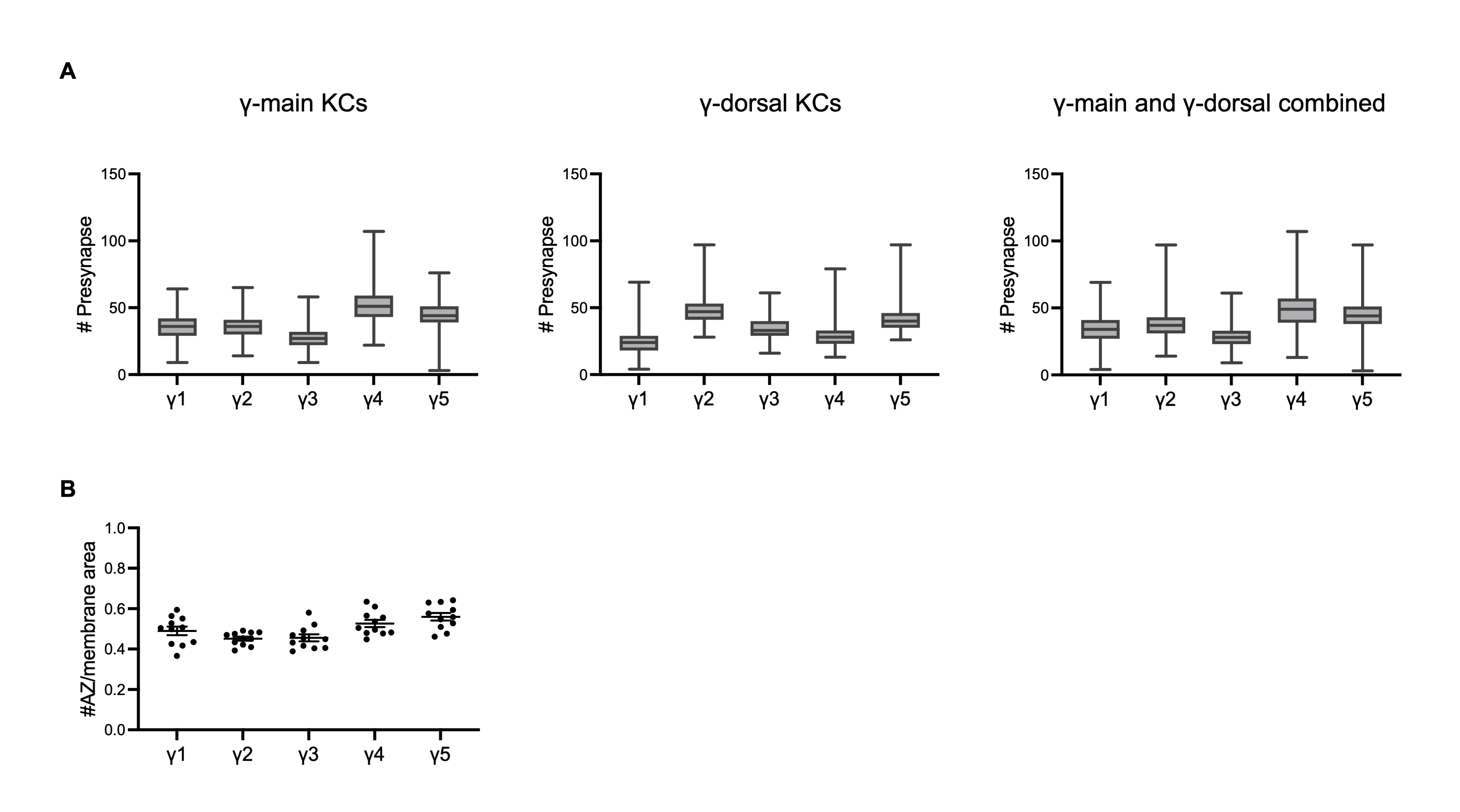

Supplement: S9 Fig — (A) Pre-synapse (AZ) number in each γ compartment annotated in the hemibrain connectome for γ-main KCs, γ-dorsal KCs and combined. All γ KCs annotated in the data set are quantified. γ-m KCs, n = 588; γ-d KCs, n = 99. Box plots showing center (median), whiskers (Min. to Max.). (B) AZ density (#Brp cluster/CD4::tdTomato area) quantified in different compartments using our image analysis pipeline. The same data set as in Fig 3B is used to quantify. n = 11 brains. Error bars show S.E.M. The data underlying this Figure can be found in S1 Data. (TIFF) [file pbio.3003449.s010.tiff]

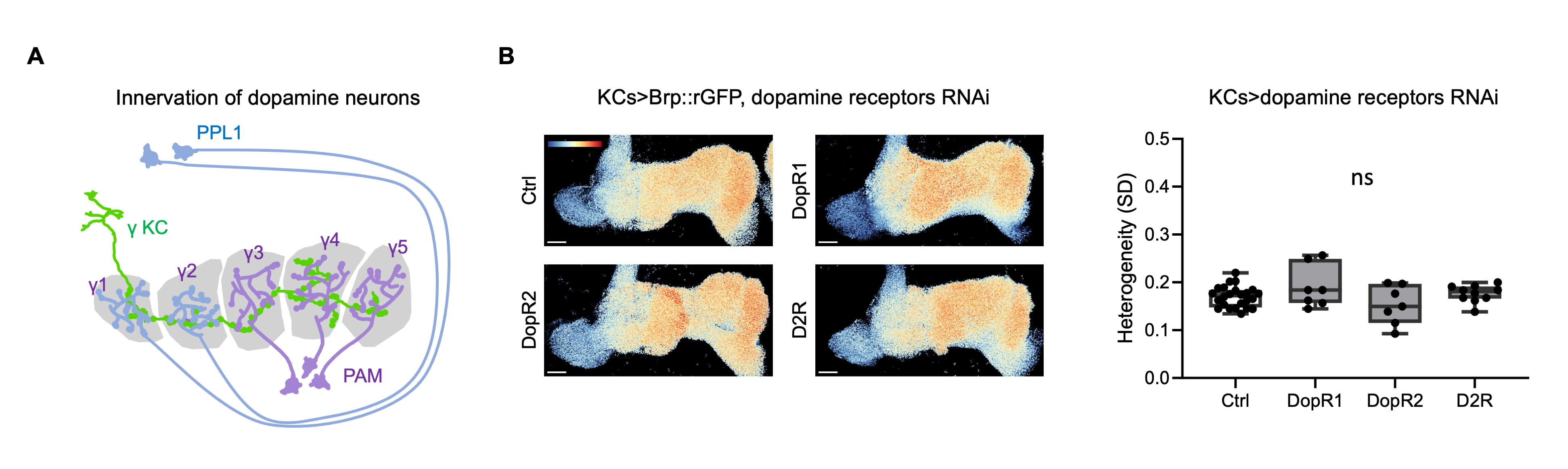

Supplement: S10 Fig — (A) Schematic showing the innervation patterns of dopamine neurons. The γ lobe is innervated by dopamine neurons from the PPL1 and PAM clusters. (B) Knockdown of dopamine receptors does not significantly alter the Brp heterogeneity level. Three types of DA receptors, DopR1, DopR2 and D2R were knocked down using RNAi in KCs specifically with R13F02-GAL4. Representative images of Brp::rGFP from each group are shown. Pseudo color in the images represents the value of log2 (pixel intensity/mean pixel intensity in the γ lobe). Pseudo color range: −1.3 to 1.3. Scale bar: 20 μm. Ctrl (n = 24) vs. DopR1 (n = 7), DopR2 (n = 7) and D2R (n = 10): P > 0.05. Box plots showing center (median), whiskers (Min. to Max.). ns = not significant by Kruskal–Wallis test. The data underlying this Figure can be found in S1 Data. (TIFF) [file pbio.3003449.s011.tiff]

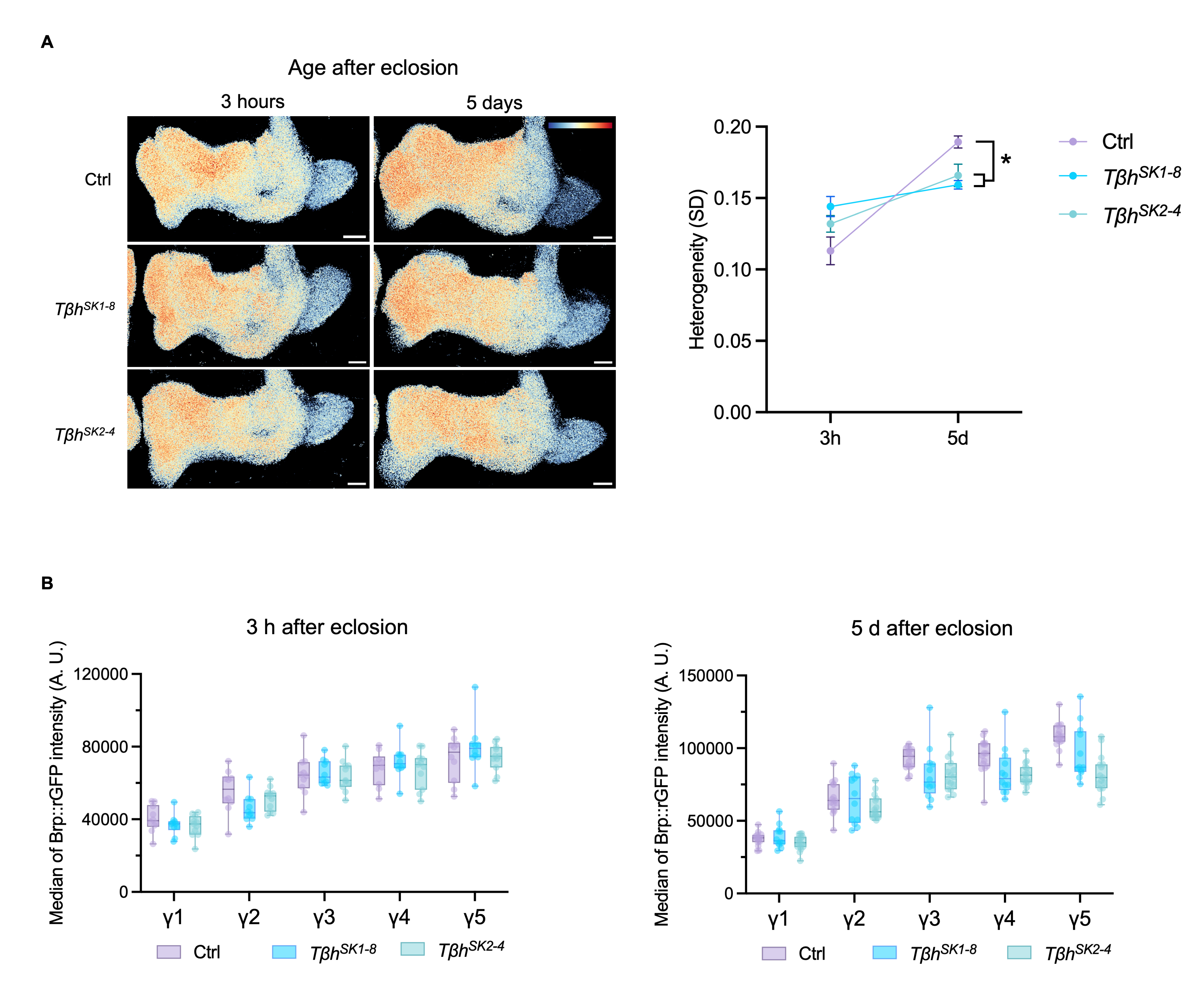

Supplement: S11 Fig — Brp::rGFP heterogeneity levels were measured at different time points after eclosion for Ctrl and Tβh mutants. Tβh mutants show impaired maturation of the compartmental Brp heterogeneity in early adulthood. (A) Brp::rGFP heterogeneity level measured at 3 hours and 5 days after eclosion. Ctrl 3 h (n = 10) vs. TβhSK1-8 3 h (n = 10): P > 0.05; Ctrl 3h vs. TβhSK2-4 3 h (n = 11): P > 0.05; Ctrl 5 d (n = 15) versus TβhSK1-8 5 d (n = 12): P = 0.0333; Ctrl 5 d vs. TβhSK2-4 5 d (n = 15): P = 0.0333. Error bars show S.E.M. Kruskal–Wallis test. *P < 0.05 and ns = not significant. Pseudo color in the images represents the value of log2 (pixel intensity/mean pixel intensity in the γ lobe). Pseudo color range: −1.2 to 1.2. (B) Median Brp::rGFP intensities in γ KCs measured at 3 h and 5 days after eclosion. Box plots showing center (median), whiskers (Min. to Max.). The data underlying this Figure can be found in S1 Data. (TIFF) [file pbio.3003449.s012.tiff]

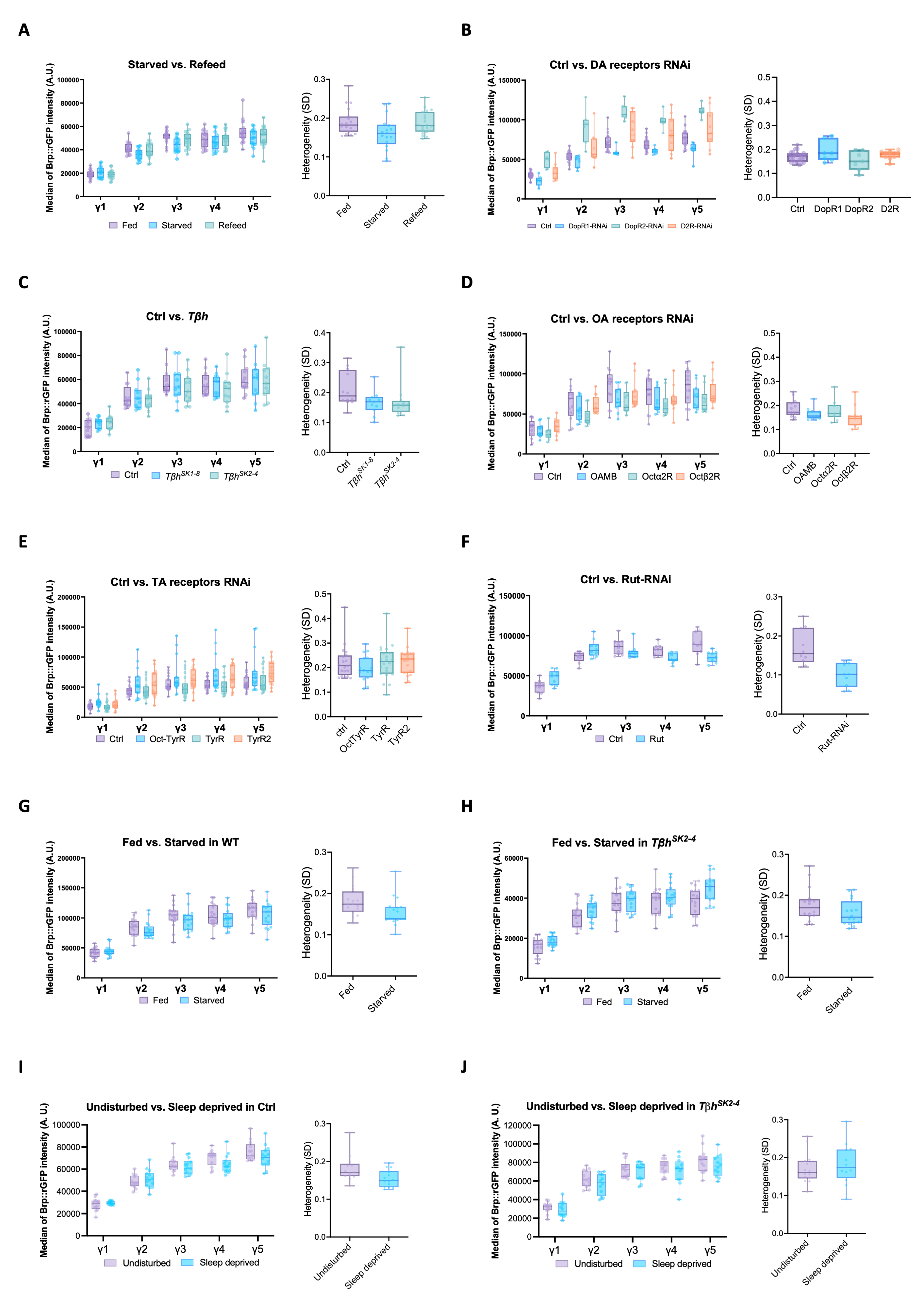

Supplement: S12 Fig — (A) Fed, starvation and refeeding in Fig 3D. (B) Dopamine receptors knockdown in S10 Fig. (C) Ctrl and Tβh comparison in Fig 4E. (D) Octopamine receptors knockdown in Fig 5A. (E) Tyramine receptors knockdown in Fig 5A. (F) Rut knockdown in Fig 5B. (G) Food starvation in Fig 6B. (H) Starvation of TβhSK2-4 mutants in Fig 6B. (I) Food sleep deprivation in Fig 6C. (J) Sleep deprivation of TβhSK2-4 mutants in Fig 6C. Box plots showing center (median), whiskers (Min. to Max.). See sample number and statistics of heterogeneity level comparison in legends of main figures. The data underlying this Figure can be found in S1 Data. (TIFF) [file pbio.3003449.s013.tiff]
