# Supplementary material for: Octopamine signaling regulates the intracellular pattern of the presynaptic active zone scaffold within Drosophila mushroom body neurons
Source: PLoS Biol. 2025 Oct 23;23(10):e3003449. doi: 10.1371/journal.pbio.3003449 (PMC12626334; doi:10.1371/journal.pbio.3003449)
Supplement: Attachment — Submitted filename: Wu Eno et al Response to Reviewers.pdf [file pbio.3003449.s014.pdf]

We thank the reviewers for their thorough and constructive feedback. In response, we have extensively revised the manuscript to address the reviewers' concerns by incorporating substantial new data and analyses. Most notably, we now demonstrate intracellular synapse heterogeneity by **quantifying clusters of endogenous Brp proteins within single Kenyon cells**. Additional new data to bolster the main conclusions and to ensure the rigor of our finding include:

- Validation of the active-zone localization and nanoscopic assembly of Brp::rGFP using **STED super-resolution microscopy**
- Verified that **split-GFP tagging does not alter Brp distribution via immunostaining**
- Demonstration of **post-eclosion maturation of Brp heterogeneity** and **the requirement of octopamine signaling (*Tβh*)**.
- **Sleep deprivation induces remodeling of the Brp distribution**, similar to feeding stress, and **octopamine signaling (*Tβh*) is required for this plasticity**.
- Confirmation of **compartmental calcium differences using a Brp-independent, membrane-tethered calcium sensor**, further supporting uneven calcium distribution with a Brp-based sensor.
- Comparison of **our imaging data to presynapse distributions extracted from the Drosophila connectome** to rule out anatomical confounds.

Finally, we revised all statistical analyses where necessary, clarified methodological descriptions, and substantially tightened the language throughout to remove imprecise or overstated claims.

Reviewer comments

Reviewer #1: The authors report neurobiological experiments using transgenic flies, Drosophila melanogaster. Drosophila represents one of the most important model organisms for studying the neuronal basis of innate and dynamically changing adaptive behavior. The authors focus on a central brain region, the so-called mushroom body, which plays a key role in orchestrating behavior based on experience (learning and memory) and internal states, such as sleep state or hunger state.

The authors used a clever technique to label the presynaptic active zone protein Bruchpilot by a fluorescent tag only in genetically determined cell groups. They found that along the axonal regions of the mushroom bodies, the so-called lobes, which are formed from bundled axons of Kenyon cells, the fluorescence is unevenly distributed. It appears that presynapses do not occur homogeneously along the axons of Kenyon cells, but more or less frequently in sub-axonal regions (compartments). The authors associate this with different levels of baseline calcium levels, which is explained by the clustering of calcium channels at the active zone. Furthermore, an extended starvation period of 2 days influences the distribution of the active zone protein Bruchpilot. This starvation-induced change does not occur when a specific octopamine receptor is downregulated. Similarly, this change does not occur when an adenylate cyclase (rutabaga) is impaired. The authors conclude a link between starvation, neuromodulation by octopamine via cAMP signaling to a change in synaptic output to adapt behavior accordingly.

The study has some interesting points. First, the technical approach of visualizing synaptic proteins by split-GFP tags in a cell type-specific manner is elegant. Second, the study of dynamic changes in synaptic proteins due to internal states of the animal is an important and interesting extension of the concepts of how neuromodulation manifests at the cellular level. Third, the study of the functioning of neuronal circuits in the Drosophila brain is the focus of current scientific interest, especially with the recent provision of the entire connectome of the Drosophila brain.

However, the study has very substantial weaknesses. In particular, none of the core statements of the study are actually covered beyond doubt by the experimental data and remain highly speculative. A list of the main concerns is given below. While the research question is of considerable interest and the imaging techniques are elegant, the manuscript suffers from substantial conceptual and experimental limitations that undermine its conclusions. Many of the key claims are speculative, poorly supported by the data, or overstated. The work is currently not ready for publication in its present form. I therefore recommend that the journal does not consider the manuscript for publication. I strongly recommend that the authors continue their research on this topic

and wait with the publication of results until the collected data allow an answer to the research question posed.

Specific points of concern:

1. The title states that "octopamine signals coordinate the spatial pattern of presynaptic machineries." In fact, it remains unclear what "coordinate" in this context actually means (upregulation, downregulation, relocation, ...?). And what exactly is "spatial pattern"? Density, amount, spatial arrangement at the active zone? It remains also unclear why the authors conclude that "presynaptic machineries" are coordinated, because they analyze only one single active zone protein and not presynaptic machineries. This is clearly an over-interpretation.

We thank the reviewer for the interest in our method and appreciate the reviewer's critique on the title. Accordingly, we have revised it to “Octopamine signals regulate the intracellular pattern of the presynaptic active zone scaffold within *Drosophila* mushroom body neurons”. We eliminated ambiguous terms, such as “coordinate” and “presynaptic machineries”. As the reviewer pointed out, our study is based on the distribution of the active zone scaffold protein Brp, but not the entire presynaptic machinery.

2. The title states that presynaptic machineries are coordinated "within individual Drosophila mushroom body neurons". Not one single experiment shows the spatial arrangement of active zone proteins in individual intrinsic mushroom body neurons (Kenyon cells). All experiments show fluorescence tags measured across large numbers of neurons and compartments in the mushroom body lobes. The claim that individual mushroom body neurons have been investigated is misleading.

We thank the reviewer for pointing out the lack of evidence from individual neurons. To directly address this concern, we have conducted experiments to analyze Brp::rGFP signals in single γ-KCs using flp-out GAL4 (Fig. 2C in the revision). The new data reveal that individual Brp::rGFP clusters in single γ-Kenyon cells vary in intensity among the compartments (Fig. 2D) in a pattern similar to a pan-Kenyon cell driver (cf. Fig. 2A and

3B). We believe this new single-cell data provides compelling evidence that individual KCs exhibit subcellular variations in active zone protein levels.

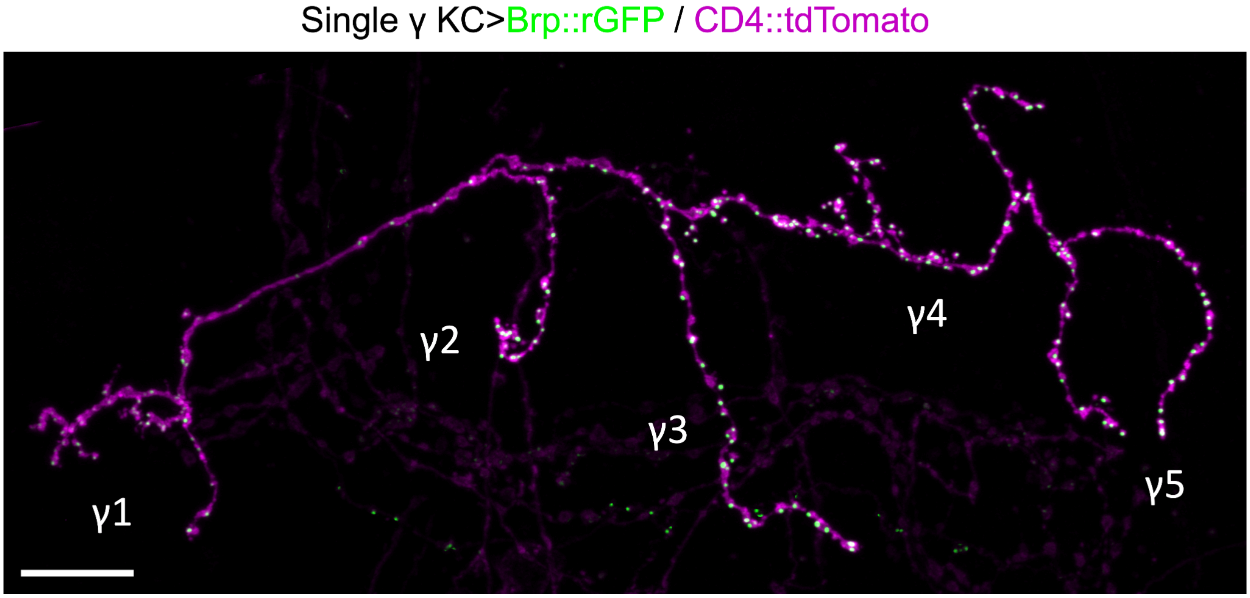

Fig. 2C Representative maximum intensity projection visualizing Brp::rGFP and CD4::tdTomato in a single γ KC. Scale bar, 10 µm.

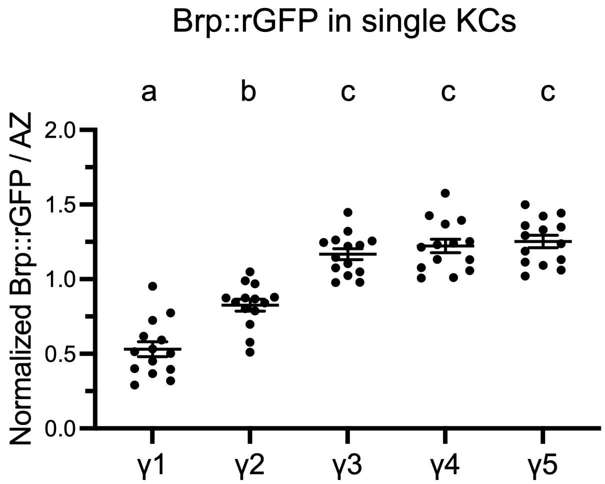

Fig. 2D Signal intensity of Brp::rGFP clusters of single KCs in different compartments. Individual Brp::rGFP clusters are measured. Significant differences ($P < 0.05$) are indicated by distinct letters. Repeated measures one-way ANOVA. See also legend in the manuscript.

3. Abstract, line 21: The first sentence "neurons need to adjust synaptic output according to the targets" makes no sense. What is meant by target? Perhaps the authors mean the postsynapse? And what exactly are target-specific synaptic

structures? If this refers to the presynaptic active zone, then the statement that these remain unresolved is simply not true. The structure and function of presynaptic active zones have been studied exquisitely and in great detail.

We thank the reviewer for pointing out the ambiguity. Accordingly, we have replaced “target” with “postsynaptic partners” in the first sentence. It has long been a common practice to use “target” to indicate the cell that makes synapse with (e.g. Schnider 2000 Neuron; Maccaferri et al., 1997 Science). We ask if a single KC might exhibit synaptic heterogeneity corresponding to different MBONs in spatially segregated compartments (Fig. 2B-D).

4. Abstract, line 26: the authors write "We found the patterned accumulation of Brp among the compartments of axon terminals,...". However, the experiments never refer to axon terminals. Instead, large bundles of axons, the mushroom body lobes, and their compartments are always examined. The abstract raises expectations in the reader that are not fulfilled by the investigations.

We consider that axon terminals typically have presynaptic boutons with the release sites of synaptic vesicles. Our single KC images show that KCs make en passant synaptic boutons within the mushroom body lobes and Brp::rGFP clusters are localized within the boutons (Fig. 2C). As previous literature has established that KCs release neurotransmitters from these sites along the lobes (Barnstedt et al., 2016 Neuron; Stahl et al., 2022 eLife), we believe “axon terminals” is an appropriate description for these regions, and we have retained this term.

5. Abstract, line 27: The authors write "...the localized octopaminergic modulation along gamma KC terminals regulate this compartmental Brp heterogeneity...". I could not find any experiment showing localized modulation, and certainly not for axon terminals. The experiments cannot rule out the possibility that octopamine acts homogeneously across all compartments. After all, octopamine can act via release from many different neurons, but also as a neurohormone.

To more accurately reflect our findings, we have replaced “modulation” with “projections”. This description is supported by anatomical evidence. There are only two octopaminergic neurons (OA-VPM3/4) projecting to the mushroom body lobes. They most densely terminate in the γ1 compartment (Busch et al., 2010 J Comp Neurol; Aso et al., 2014 eLife). We have confirmed this result using Brp::rGFP in these neurons (Fig. 4). As such, we believe that γ1 could receive more octopaminergic modulations when OA-VPM3/4 are activated.

6. Abstract, line 30: The authors write: "Such coordinated regulation....explains how the mushroom bodies integrate changing physiological states." The term "explains" is really overstated; at best, it can suggest a possible mechanism. The experiments described do not explain the functioning of the mushroom body, which necessarily manifests itself as a function of a neuronal circuit. In fact, the function of the neuronal circuit is not even the subject of this study.

We acknowledge that the word “explains” might be overstating, and replaced it with “suggests”.

7 Abstract, line 31: What exactly is the "computational capacity of the circuit"? How is it measured and why does it need to be expanded? And why do the results of the reported experiments on the visualization of Brp indicate that the computational capacity of the circuit is expanded?

By “computational capacity” we refer to the circuit’s ability to transform inputs to diverse, compartment-specific outputs. In the *Discussion* section, we propose that the subcellular synaptic heterogeneity could increase the output diversity of KCs to differentially control MBONs. We have deleted the related sentence in the abstract.

8. Introduction, line 35: The molecular compoisition of synapses is certainly not the only factor that determines the function of neurons.

We agree with the reviewer that it is not the only factor and have no intention to say it is. To avoid confusion, we therefore replaced “determines” with “underlies.”

9. Introduction, line 42: What exactly is meant by "target-specific AZ regulation"? If it means that the active zones and the postsynapses are altered in a coordinated manner, then the statement that this has been "scarcely studied" is simply wrong. There is an enormous number of publications investigating the regulation of active zones.

As stated in the response to the reviewer’s point 3, “target” here referred to the postsynaptic MBONs. To clarify our intention, we have included a citation to a review (van Oostrum and Schuman, Nature Review Neuroscience 2025) which mentions the heterogeneous presynaptic protein composition according to postsynaptic cells (cf. Fig. 1 in the review above).

10. Introduction, line 56: What exactly is meant by "estimating the synapse function"? And what about Brp structures? Possibly the authors mean that the number of Brp molecules at a presynapse can give an indication of the amount of vesicles to be released, i.e. can help determine the efficiency of synaptic transmission. If the authors mean this, they should formulate it precisely.

We have replaced “estimate synapse function” with “Brp enrichment can serve as a proxy for estimating the synaptic vesicle release probability of single AZs” and included the citation (Akbergenova et al., 2018 eLife; Newman et al., 2022 Nature Communications).

11. Introduction, line 59: What do the authors mean by "profiling the character of individual Brp clusters"? The experiments only provide information about the relative amount of Brp in compartments of MB lobes. It is not clear what is meant by "character" or "profiling" of the same.

“Profiling the character of individual Brp clusters” here means comprehensive quantification of individual Brp::rGFP clusters in defined cell types, such as intensity and density. We have clarified our image analysis pipeline by adding a graphical explanation (Fig. 3A). We report detailed validation of cluster detection in Wu et al., 2025 eLife Reviewed Preprint.

12. Results: The approach of visualizing Brp via split-GFP is very good and clever. However, the manuscript lacks studies on whether the GFP11 tag affects the localization or function of Brp in any way or modifies the amount of Brp. In this context, it is important to indicate whether the animals studied are homozygous or heterozygous for the Brp gene tagged with GFP11. Statements about the influence of the tag on the function or distribution of Brp can of course be better tested in homozygous animals. In heterozygous animals, in which an unknown high proportion of Brp is visualized, there is always the risk that wild-type and tagged Brp are differentially localized, i.e. the unequal distribution does not reflect the natural conditions.

We thank the reviewer for raising the essential point on our visualization strategy. We here used heterozygous animals for *brp::GFP*$_{11}$ and included this information in the text (see also Methods section).

Regarding the Brp localization, A large body of studies using various strategies has validated that the N-terminal region and coiled-coil domains, but not the C-terminal, region of Brp are necessary and sufficient for active zone localization (Fouquet et al., 2009 J Cell Biol; Oswald et al., 2010 J Cell Biol; Mosca and Luo, 2014 eLife; Kiragasi et al., 2017 Cell Rep; Akbergenova et al., 2018 eLife; Nieratschker et al., 2009 PLoS Genet; Johnson et al., 2009 PLoS Biol; Hallermann et al., 2010 J Neurosci).

To directly validate active-zone localization of Brp::rGFP, we present multiple lines of evidence. First, we performed STED microscopy for the larval motor neuron and confirmed the donut-shape arrangement of Brp::rGFP (Fig. S3), corroborating the assembly of Brp::rGFP at presynaptic active zones.

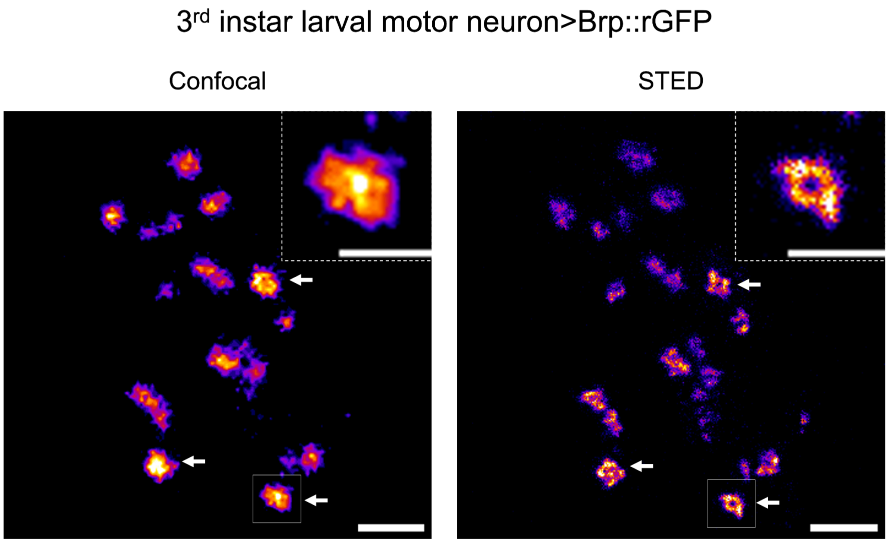

Fig. S3 Confocal and STED images of Brp::rGFP in 3rd instar larval motor neuron terminals. Scale bar, 1 μm in the overviews; 500 nm in the insets.

Second, we have also compared anti-Brp staining between wild type (*Canton-S*) and *brp::GFP*$_{11}$/+, and found indistinguishable patterns (Fig. R1). Furthermore, the numbers of Brp::rGFP clusters we counted are comparable to the pre-synapse numbers in the connectome data (cf. Fig. 1E in Wu et al., 2025 eLife Reviewed Preprint). These results provide additional evidence that rGFP signals in the heterozygous animals accurately report endogenous Brp.

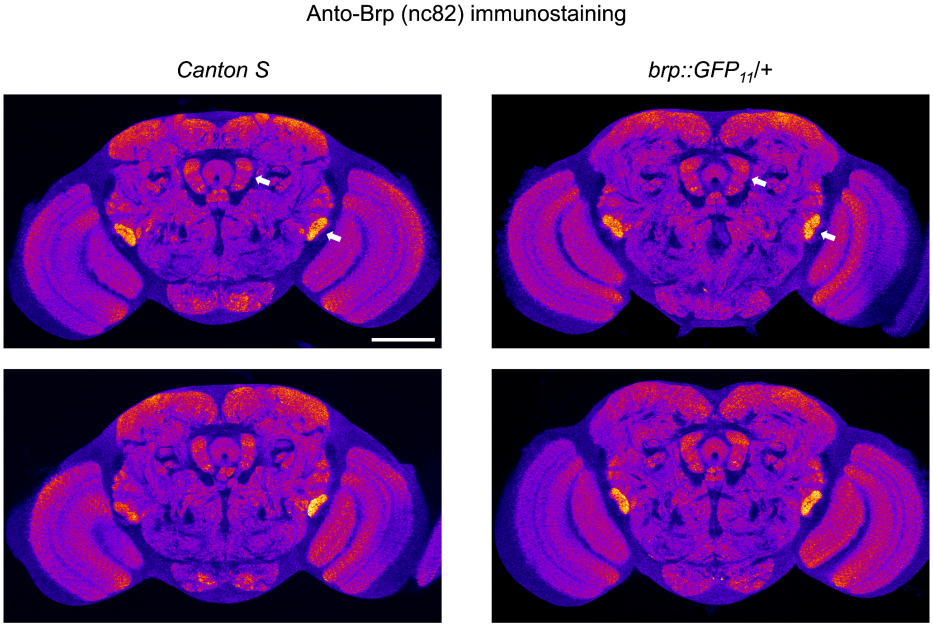

Fig. R1 Anti-Brp (nc82) immunostaining for Canton S and *brp::GFP$_{11}$*/+ fly brains. White arrows indicate brain areas where Brp signals show similar patterns between the two groups. Scale bar, 100 μm.

13. Results, line 75: If the authors claim that the puncta match the axon terminals, then double staining of Brp puncta and axon terminals must be performed to prove the match.

The location of presynaptic terminals of these cell types have been characterized in previous studies (for dopamine neurons, Mao and Davis, 2009 Frontiers Neural Circuits; Aso et al., 2010 Current Biol; Liu et al., 2012 Nature; for APL and DPM neuron, Liu et al., 2009 Nat Neuroscience; Pitman et al., 2011 Current Biol; Haynes et al., 2015 eLife; Chu et al., 2019 Nature Comms). Furthermore, presynaptic sites of these neurons have been annotated in the connectome data (Scheffer et al., eLife 2020). We thus believe Brp::rGFP is localized to the axon terminals and kept this term.

14. Results, line 78: The authors write: "...these results suggest disinct accumulations of Brp intracellularly". This is a very central point in the argumentation, but it is not shown experimentally. In order to demonstrate the intracellular distribution of Brp, it is necessary to stain individual Kenyon cells. This is a major caveat of the present study. Although it is claimed that Brp is unevenly distributed in individual Kenyon cells, the fluorescence measurements only cover large populations of axons. Considering that even individual gamma-obe Kenyon cells show more axon collaterals the closer the axons get to the midline, the more logical it is that more and more presynapses occur along the lobes. Another problem is that the images represent one focal plane, but the MB lobes are three-dimensional, curved structures. It is quite possible that in Figure 1B or 2A, for example, the first compartment of the gamma lobes does not lie in the focal plane and therefore has a weaker fluorescence signal.

We thank the reviewer for raising these critical points regarding the interpretation of our data. Following the reviewer's comment, we analyzed Brp::rGFP in single γ-KCs using flp-out GAL4 to drive GFP$_{1\text{-}10}$ expression stochastically (Fig. 2C). This approach allows unambiguous isolation of Brp clusters and revealed substantial variability in Brp::rGFP

cluster intensity among the compartments (Fig. 2D). This intracellular pattern of Brp intensity was similar to the one with a pan-Kenyon cell driver (e.g. Fig. 2A and 3B).

Regarding the alternative explanation that our results simply reflect changing synapse density, it is important to note that the quantification of Brp::rGFP signal intensity in this study is based on individual clusters and therefore independent of the cluster density or numbers in compartments (please also see our response to Point 3). To substantiate this, we have analyzed the connectome data and found that the pre-synapse number in each compartment does not explain the Brp heterogeneity (Fig. S9). Likewise, the density and intensity of Brp::rGFP clusters over the compartments were different (cf. Fig. S9B; Fig. 3B and Fig. S12).

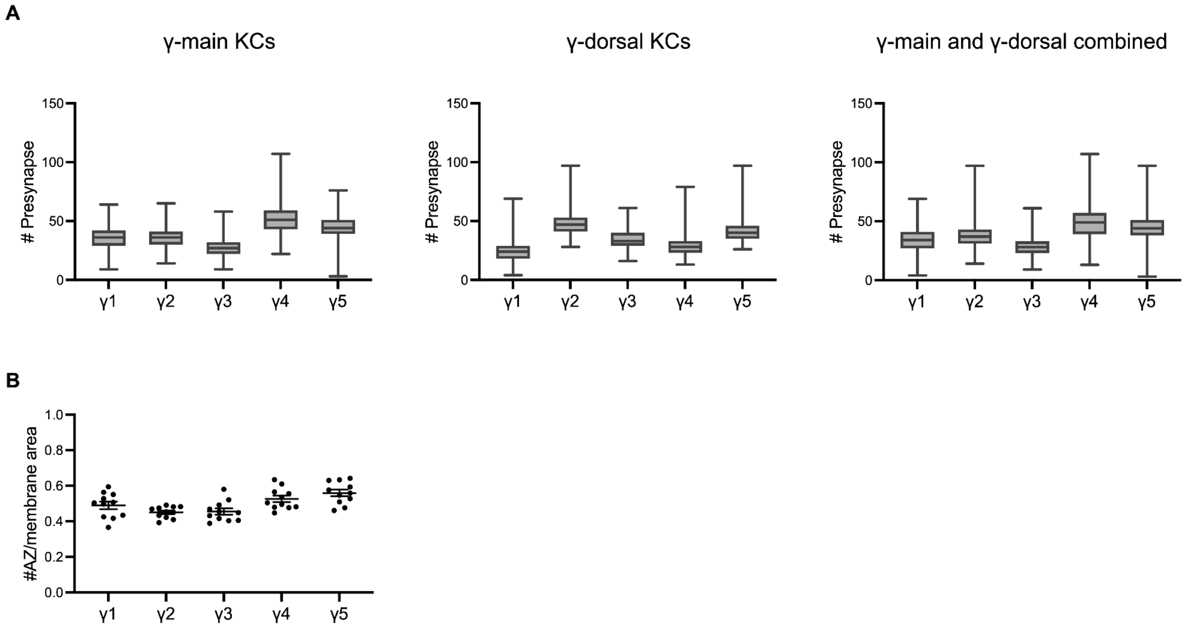

Fig. S9 AZ number/density difference along γ lobe compartments. (A) Pre-synapse (AZ) number in each γ compartment annotated in the hemibrain connectome for γ-main KCs, γ-dorsal KCs and combined. All γ KCs annotated in the data set are quantified. γ-m KCs, n = 588; γ-d KCs, n = 99. Box plots showing center (median), whiskers (Min. to Max.). (B) AZ density (#Brp cluster/CD4::tdTomato area) quantified in different compartments using our image analysis pipeline. The same data set used for Fig. 3B is quantified. n = 11 brains. Error bars show S.E.M.

We apologize for the confusion regarding image processing for representative photos, such as Fig. 1B and Fig. 2A, and have clarified in the legends that they are maximum intensity projections. It is a legitimate concern on weak signals due to depth in 3D volume, since the γ1 compartment is typically located deeper in the sample. We controlled it using samples where the γ1 and γ5 compartments happened to be on the

same optical slice (depth) and still found weaker intensity in the γ1 compartment for Brp::rGFP, but not CD4::tdTomato, signals (Fig. S5). These new data and analyses fully address the reviewer's concern.

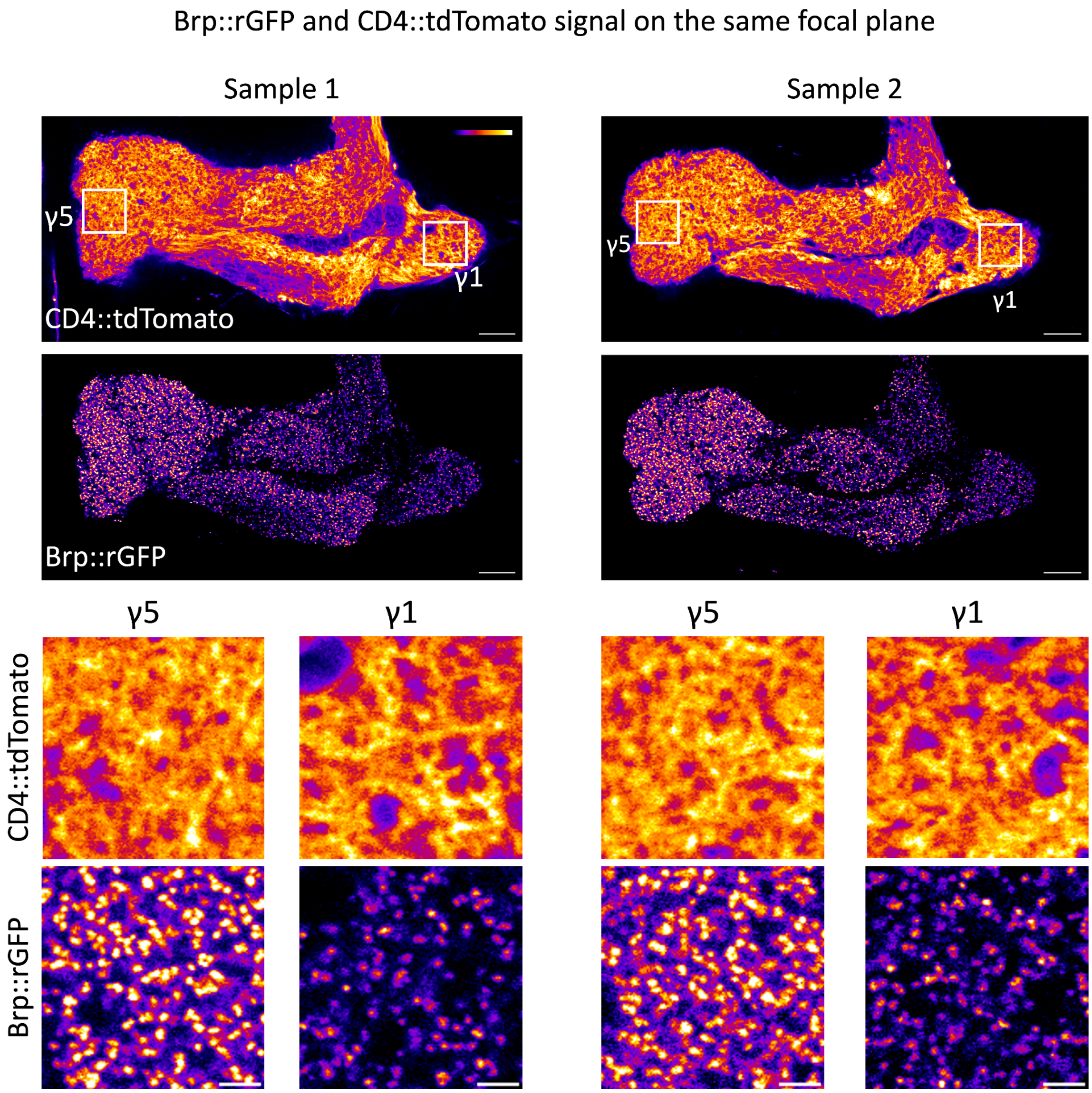

Fig. S5 CD4::tdTomato and Brp::rGFP in γ5 and γ1 imaged on the same focal plane. Upper panels, single slice in the image stack where both γ5 and γ1 are shown. White boxes indicate zoomed-in areas shown in the lower panels. Scale bar, upper panels: 10 μm; lower panels: 2 μm. See legend in the manuscript also.

15. Result, line 90: It is important to point out that the active zone heterogeneity among MB compartments has not been determined at the single cell level. Rather, the authos have measured fluorescence intensities across large groups of neurons. The headline

"Intracellular" active zone heterogeneity is therefore misleading. The data suggest only that the numbers of presynapses increase over the length of the axon bundles.

This concern has been addressed through the inclusion of the single-KC analysis (Fig. 2C-D), which clearly shows the intracellular heterogeneity in Brp clusters.

16. Results, line 99: The authors write that "individual KCs tune their presynaptic structures according to compartments." First, the experiments have not been conducted on individual KCs. Second, the data shown in figures 2B indicate something else: all compartments show actually the same fluorescence intensity, except for the gamma 1 compartment. In line 108, the authors then write "this compartmental pattern of GCaMP signal nicely mirrored the Brp heterogeneity". This I cannot see in the data. Whereas figure 2B shows that only the gamma 1 compartment differs from the other compartments, figure 2D shows a slight, gradual increase in GCaMP/mCherry fluorescence. The results do not match at all.

We thank the reviewer for careful inspection of our data and for pointing out that our description could be clearer. We have reanalyzed the data in previous Fig. 2B (now Fig. 3B) and Fig. 2C (now Fig. 2E) using the more appropriate repeated one-way ANOVA as suggested by the reviewer (i.e. points 18 and 22). This analysis revealed multiple significant differences, beyond a simple distinction at the γ1 compartment (Fig. 2D-E). We now report the similarity of Brp::rGFP and GCaMP/mCherry ratio compartmental patterns using more factual and accurate description.

17. The authors want to quantify basal calcium levels and use a red fluorescent tag (mCherry) of Brp for normalization. However, it is not clear whether the red and green fluorescence (mCherry and GCaMP) change equally linearly when the amount of Brp is changed. It would be much better to use a calcium sensor to measure basal calcium concentrations that is independent of Brp, e.g. Campari, especially since the amount and possibly function of Brp has been artificially manipulated by the ectopic expression of the Brp:mCherry:GCaMP construct.

We have addressed this point by re-analyzing the linearity of mCherry and GCaMP fluorescence (Fig. R2). Intensities of red and green fluorescence vary considerably, but show strong correlation in all compartments (Fig. R2).

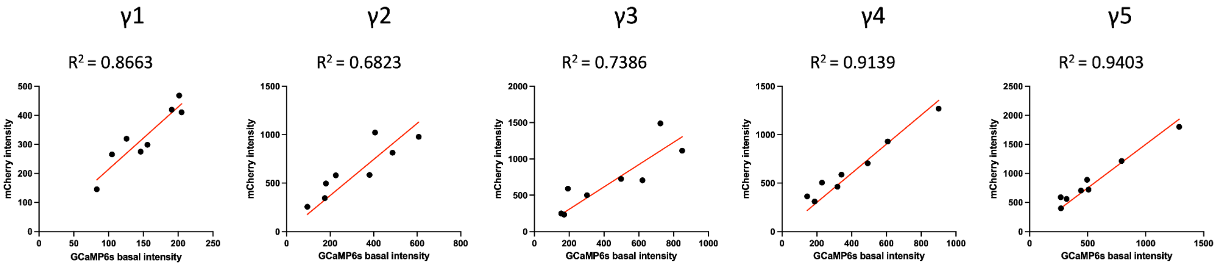

Fig. R2 Correlations between mCherry and GCaMP fluorescence level in individuals expressing different amounts of UAS-Brp::mCherry::GCaMP6s. Same data set as in Fig. 2E. Each data point represents one individual. n = 8 animals. The square of Pearson R is calculated.

Following the reviewer’s suggestion, we have also conducted the same measurement using a Brp-independent Ca^{2+} sensor, myr-GCaMP6s (Akebergenova et al., 2018 eLife) with CD4::tdTomato as a reference, and found the pattern similar to those of Brp::mCherry::GCaMP and Brp::rGFP intensity (Fig. S8). The gradual increase of basal calcium from γ1 to γ5 is thus shown by these two distinct sensor systems, resolving the reviewer’s concern on the Brp dependency and fluorescence linearity.

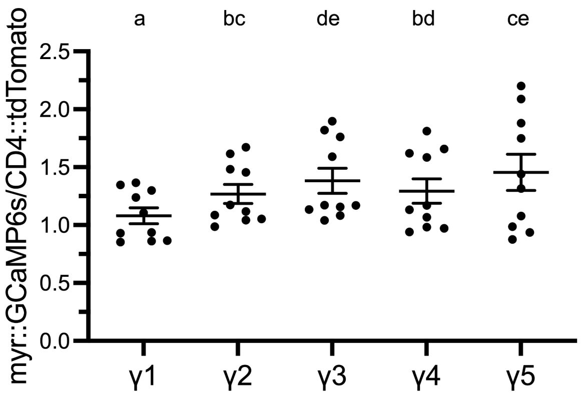

Fig. S8 Basal Ca2+ concentrations in different γ compartments measured by myr::GCaMP6s. The ratio of myr::GCaMP6s to CD4::tdTomato in γ compartments is shown. Experimental procedures and quantification are comparable to that of Fig. 2E. Error bars show S.E.M. Significant differences ($P < 0.05$) are indicated by distinct letters. Repeated measures one-way ANOVA.

18. In Figure 2, fluorescence values are statistically compared between MB compartments. However, it is not indicated which posthoc pairwise test was applied. If the measurements between the compartments were made in the same preparations (which would be logical), then the Kruskal-Wallis test is incorrect as it requires independent samples.

We appreciate this important correction. We have reanalyzed all relevant data using repeated-measures one-way ANOVA where appropriate, replacing Kruskal-Wallis tests that assume group independence.

19. Results: In figure S2, the authors compare the numbers of presynapses between MB lobes, as it is published in an online connectome database. One can actually quantify the numbers of presynapses in this database for distinct MB compartents. This would indicate whether the actual number of presynapses in the distinct gamma 1 compartment are lower than in the rest of the gamma lobe, as the fluorescence values (figure 2A) and perhaps the calcium imaging data might suggest.

Following the reviewer's recommendation, we have analyzed compartment-specific presynapse numbers of γ KCs using the hemibrain connectome dataset (Scheffer et al., eLife 2020). We found that Brp::rGFP heterogeneity does not correlate with pre-synapse number (Fig. S9). We have further analyzed the density of Brp::GFP clusters, which neither explained the compartmental pattern of Brp intensity (Fig. S9). Please also see our response to Point 14.

20. Results, line 120: The authors write that "acute stress can drive local adjustment of AZ structures within KCs". I do not follow this conclusion. The authors test hunger, not acute stress.

We have revised the text to "feeding stress." To further address this point in science, we asked if this Brp remodeling is specific for feeding stress and conducted new experiments to examine the effect of sleep deprivation on the Brp heterogeneity.

Twelve-hour sleep deprivation caused reduction in Brp heterogeneity similar to food deprivation, and this plasticity required *TβH* (Fig. 6C). Since two distinct physiological stressors induce a similar synaptic remodeling via a common pathway, the conclusion that physiological stress reorganizes intracellular Brp accumulation patterns in KCs is well-supported.

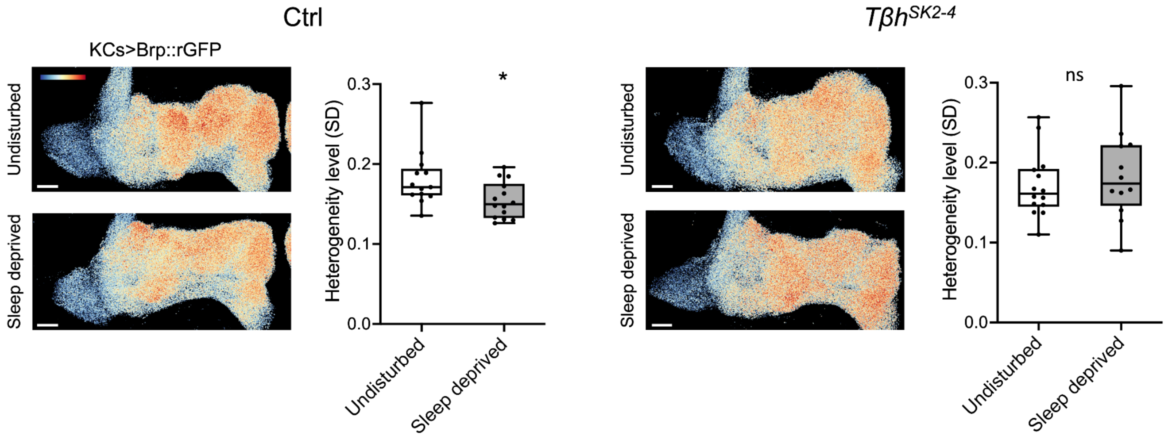

Fig. 6C Sleep deprivation for 12 hours decreases Brp::rGFP heterogeneity level in a *Tβh*-dependent manner. See legend in the manuscript also.

21. The authors claim that knocking down dopamine receptors does not affect the accumulation of Brp in MB compartments. Statistical tests for figure S7 are not indicated. However, the data shown in figures S7B indicate actually extremely strong differences between control groups and RNAi lines. In fact, the effects look much stronger than for oct beta 2R (figure S8D), including the variability that must underlie the calculation of heterogeneity. In this context, it is also not clear why in this case a log transformation of median dat has been conducted and why this data transformatio step was considered to be necessary/justified.

The statistical test used in Fig. S7B was indicated at the end of the legend in the original version. While dopamine receptor knockdown (especially *Dop1R2*) altered overall Brp levels, it did not affect the relative distribution (heterogeneity) across compartments. Log transformation was applied to measure heterogeneity levels in fold changes (i.e. relative distribution) across compartments, rather than the differences in absolute levels. Therefore, dopamine receptor knock-down did not significantly alter the compartmental

ratio of Brp::rGFP intensity. Regulation of absolute Brp levels was investigated elsewhere (e.g. Gilestro et al., 2008 Science; Gupta et al., 2016; Zhang et al., 2018 Neuron; Huang et al., 2020 Current Biol; Weiss et al., 2021 Current Bio; Huang et al., 2022 PLoS Biol; Turrel et al., 2022 Current Biol; Weiss et al., 2024 PNAS), but not the subject in this study.

22. Results, line 140: In TbetaH mutants, the authors found a decreased compartmental heterogeneity of Brp-tagged fluorescence. They conclude that octopamine controls AZ structure in KCs. However, in the respective figures (fig. 4E and S8C) one can ony see an effect in the gamma 1 compartment, which seems to show a brighter fluorescence. Overall, we do not get any indication whether any manipulation (starvation, mutation of TbH, rutabaga knockdown) increases Brp expression in gamma 1 or decreases it in gamma 2-5. This information would be important to interpret the decrease in heterogeneity. In figure 4 E, the Kruskal-Wallis test is incorrect if the compartments have been compared within the same animals. The statistical tests for pairwise posthoc comparisons are missing throughout the entire manuscript.

Our study was designed to measure the relative pattern of Brp distribution (i.e., heterogeneity) across γ compartments, rather than the absolute expression level in any single one (please also see response to the previous point). We agree with the reviewer that the Kruskal-wallis test is not suitable for data in the original Fig. 2, Fig. 4C and Fig. S1, and have reanalyzed using repeated measures one-way ANOVA. For other data, where we compare heterogeneity levels, we kept the Kruskal-Wallis test, if data were non-normally distributed and groups were independent. The original False Discovery Rate (FDR) method of Benjamini and Hochberg correction is used for all post hoc multiple comparisons (see *Statistical analysis*). Corrected P values were reported in the figure legends.

23. Discussion, line 164: The authors claim that they "resolved individual AZs at the single-cell level". For the main results concerning Kenyon cells of the MB, this is not true. No experiment at the single-cell level is reported.

As noted above, new experiments analyzing Brp distribution in single γ-KCs (Fig. 2C-D) now provide strong evidence for intracellular heterogeneity at the single-cell level.

In conclusion, this study addresses a timely and interesting question using innovative genetic labeling tools. However, substantial improvements are necessary to validate the experimental results, strengthen statistical rigor, and tone down speculative claims. I encourage the authors to refine their experimental design, increase cellular resolution, and revisit their interpretations.

We have addressed all these points by clarifying our language, including new data to validate our method and results, improving statistical tests and strengthening data interpretations.

Reviewer #2: Motivational context is a strong driver of memory acquisition, retrieval, and arousal state across species and assays. In this study, the authors begin to examine the role of hunger-dependent release of the neuromodulator octopamine (OA) on shaping synaptic protein distribution in the fly Mushroom body (MB), a key memory center. Using a novel genetic strategy, Wu et al specifically label pre-synaptic Brp in MB Kenyon cells that project across sequential axonal compartments. They find that Brp abundance varies between compartments of the gamma lobe, and that this compartment heterogeneity is disrupted in mutants for the OA biosynthesis gene TBh and by food deprivation. These results provide a promising approach to examine the impact of neuromodulation and/or experience on shaping the abundance and distribution of key synaptic proteins in the MB. The studies are generally well controlled, clearly described, and use a new, precisely targeted genetic approach. A few comments listed below can be addressed to further strengthen the manuscript.

Specific Comments

1. The influence of OA signaling on Brp distribution across gamma lobe compartments is very interesting. Is there evidence to indicate whether this

phenomenon is influenced by development or adult experience? Measuring the impact of OA disruption via TBh mutations in newly eclosed flies may be one strategy to test whether Brp distribution is impacted during development. Acutely silencing OAergic neurons in adult flies would be a clear experiment to test the role of OA in maintaining heterogeneity of Brp across compartments.

In response to reviewer's suggestion, we have conducted new experiments to compare Brp heterogeneity in newly eclosed *Tβh* mutants. This experiment led to several findings: heterogeneity levels of Brp::rGFP increase after eclosion in control KCs; *Tβh* mutants showed less heterogeneity levels at days, but not within 3 hours, after eclosion (Fig. S11). Together with the requirement of *Tβh* for stress-induced plasticity (Fig. 6), these results suggest experience-dependent intracellular distribution of Brp in the adult stage.

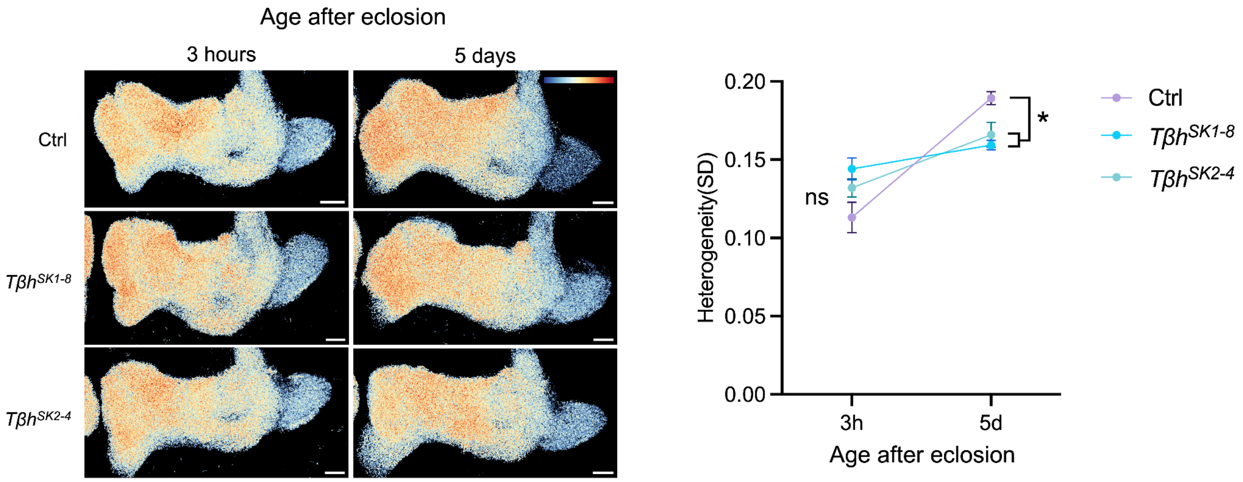

Fig. S11A Maturation of compartmental heterogeneity of Brp clusters in adult KCs requires *Tβh*. Brp::rGFP heterogeneity levels were measured at 3 hours and 5 days after eclosion for Ctrl and *Tβh* mutants. Error bars show S.E.M. Kruskal-Wallis test. *P < 0.05 and ns = not significant. See also legend in the manuscript.

2. Does altered Brp distribution with OA disruption or starvation reflect modifications in active zone size or number in each compartment? Resolving individual active zones may be technically difficult but some discussion of the underlying synaptic changes would be informative for the reader. Drawing correlations between Brp abundance in each compartment and KC synapse count from connectome datasets might provide additional insight here.

To determine if changes in active zone number could explain our results, we have performed several new analyses as suggested. We have first analyzed the pre-synapse number of γ KCs in the hemibrain connectome dataset (Scheffer et al., eLife 2020). The pre-synapse number difference among compartments does not explain the Brp heterogeneity (Fig. S9). We have further analyzed the density of Brp::GFP clusters, which neither explained the compartmental pattern of Brp intensity (Fig. S9). These data provide evidence that the Brp heterogeneity we describe reflects changes in the amount of scaffold protein per active zone, not a change in active zone number or density.

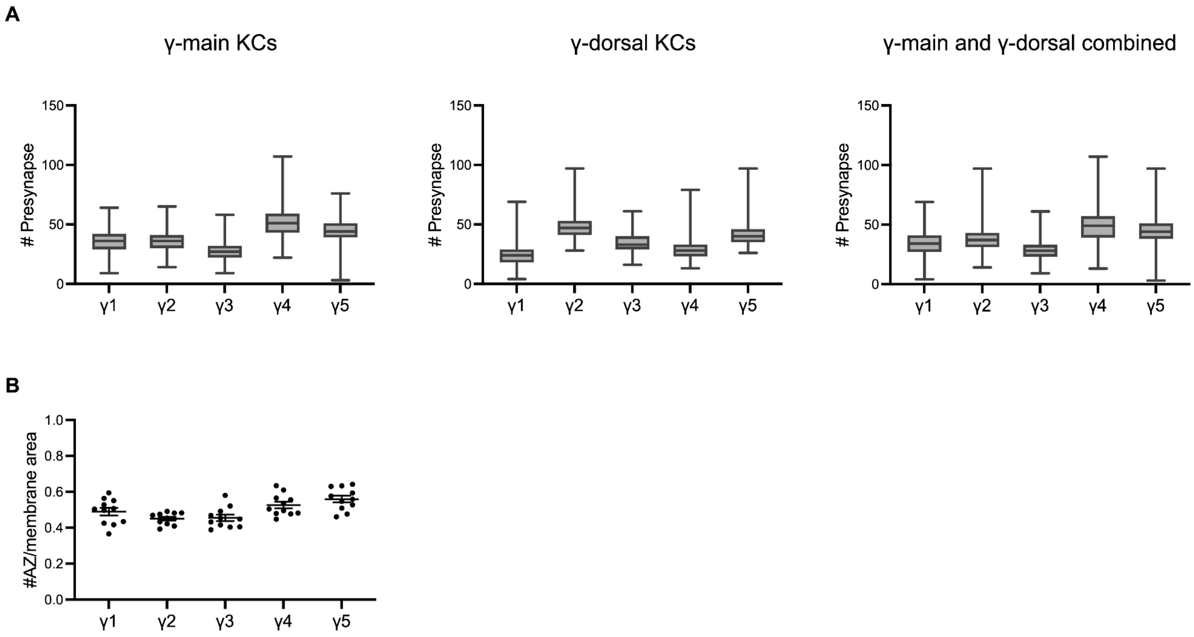

Fig. S9 AZ number/density difference along γ lobe compartments. (A) Pre-synapse (AZ) number in each γ compartment annotated in the hemibrain connectome for γ-main KCs, γ-dorsal KCs and combined. All γ KCs annotated in the data set are quantified. γ-m KCs, n = 588; γ-d KCs, n = 99. Box plots showing center (median), whiskers (Min. to Max.). (B) AZ density (#Brp cluster/CD4::tdTomato area) quantified in different compartments using our image analysis pipeline. The same data set as in Fig. 3B is used to quantify. n = 11 brains. Error bars show S.E.M.

Furthermore, we have compared the Brp cluster density between WT and *Tβh*$^{SK2\text{-}4}$ and found no significant difference (Fig. R3). Accordingly, we revised the text to describe that compartmental heterogeneity based on the intensity of individual Brp::rGFP clusters is not affected by active zone density/number.

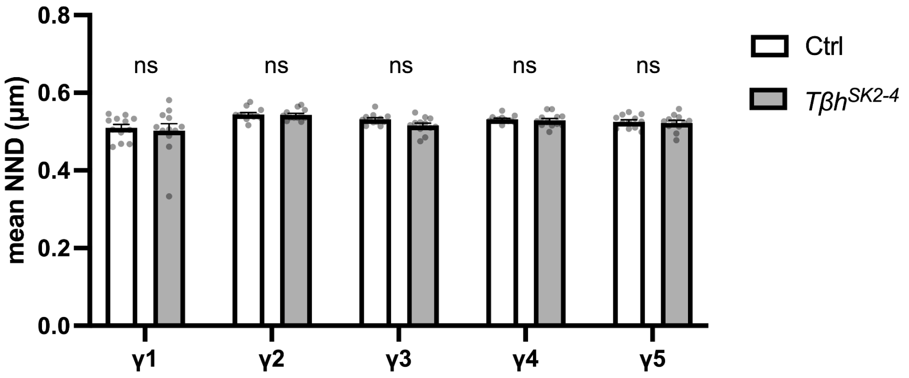

Fig. R3 Mean nearest neighbor distance (NND) of Brp::rGFP clusters in the control and *Tβh* mutants. Multiple Mann-Whitney test with FDR (BH), n = 12 for both groups.

Reviewer #3: This manuscript by Wu and colleagues describes how octopamine signals in the mushroom body alter compartmentalized synaptic structure during starvation at the synaptic level. The study uses a novel approach to label numerous individual synapses in densely-innervated structures in a genetically-targeted manner, allowing quantification of synaptic densities and analysis of synapse composition (e.g., Brp concentration). The major conclusion is that octopaminergic circuits mediate a change in synaptic composition, which can be visualized as a change in Brp signal homogeneity, during starvation that is reversed upon refeeding. The study will provide a valuable addition to the field. There are three points that should be addressed to bolster the interpretations, as described below.

1) Quantification of the Brp signal relies on signal intensity. This makes sense, though there is a caveat when comparing across compartments. Signal of various non-synaptically-localized fluorphores (e.g., cytosolic GCaMP) is also often lower in gamma1. This raises the question of whether the lower measured intensity of Brp::rGFP clusters in gamma1 is due to a technical issue (e.g., tissue depth) rather than lower expression at the synapse. This should be addressed, either with experiments to support the interpretations or reworking the interpretations.

We thank the reviewer for raising this important technical point. We have included new data comparing to show that the imaging depth does not explain the Brp heterogeneity (Fig. S5). We specifically analyzed preparations where the γ1 and γ5 compartments

were located on the same focal plane. In these samples, Brp::rGFP intensity was consistently lower in γ1, while a co-expressed membrane marker (CD4::tdTomato) showed similar intensity across both compartments. Other data such as calcium imaging is controlled by calcium-insensitive fluorophores at the same tissue depth (e.g. Fig. 2E).

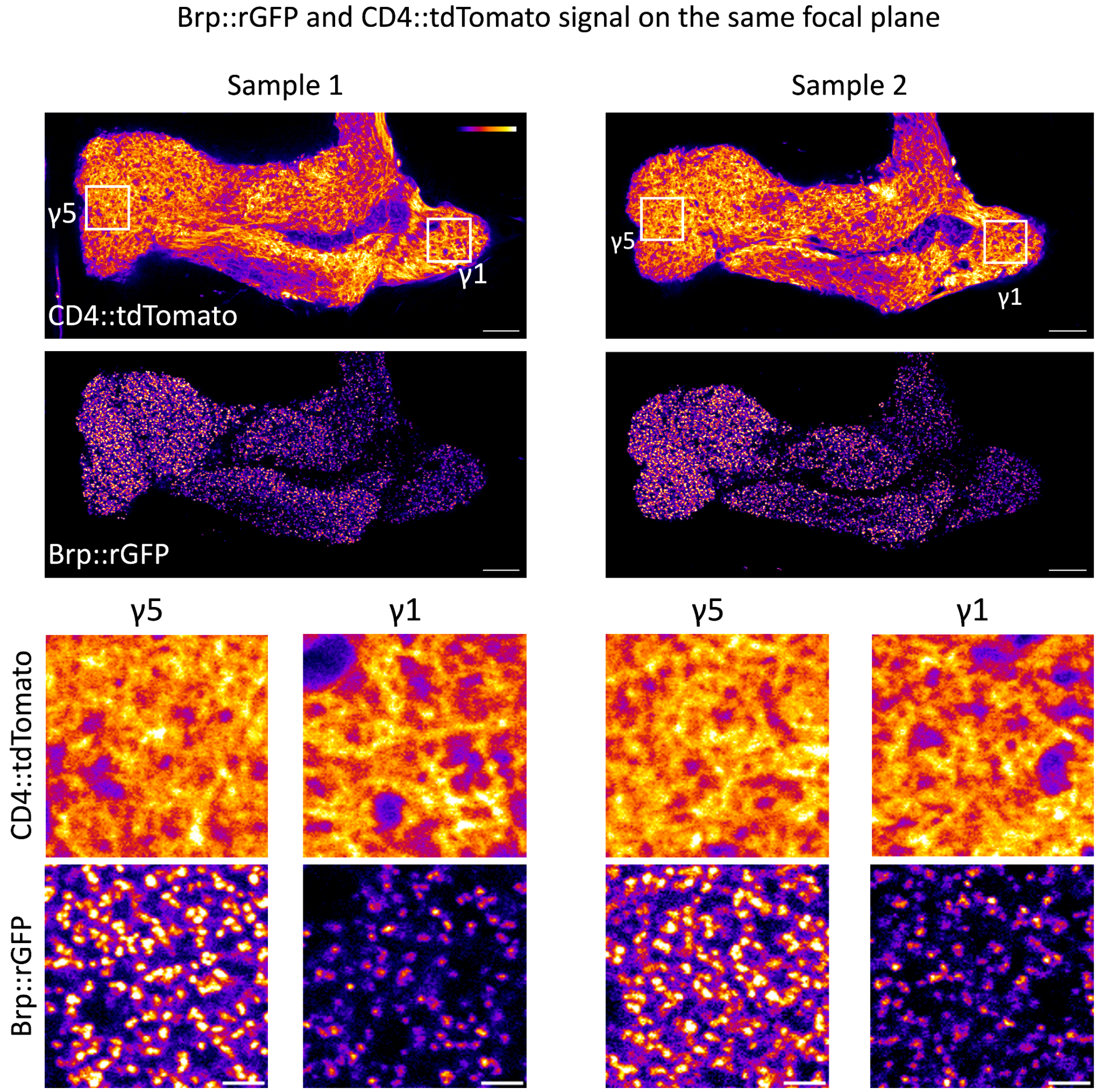

Fig. S5 CD4::tdTomato and Brp::rGFP in γ5 and γ1 imaged on the same focal plane. Upper panels, single slice in the image stack where both γ5 and γ1 are shown. White boxes indicate zoomed-in areas shown in the lower panels. Scale bar, upper panels: 10 µm; lower panels: 2 µm. See legend in the manuscript also.

2) A control experiment is necessary for Fig. 2D to ensure that the differences across compartments are due to Ca2+ concentrations (given that steady-state measurements of fluorescence from single-wavelength Ca2+ indicators are subject to various potential

artifacts). Several options would include using a Brp::mCherry::GFP, imaging the Brp::mCherry:GCaMP6s in fixed (i.e., dead) brains, or using functional imaging to examine evoked Ca2+ influx rather than basal Ca2+.

We thank the reviewer for suggestions for control experiments to validate our basal calcium measurements. We have conducted experiments using a Brp-independent Ca2+ sensor, myr::GCaMP6s (Akerbergenova et al., 2018 eLife) with CD4::tdTomato as a reference, and found the pattern is similar to that of Brp::mCherry::GCaMP6s and the Brp heterogeneity (Fig. S8).

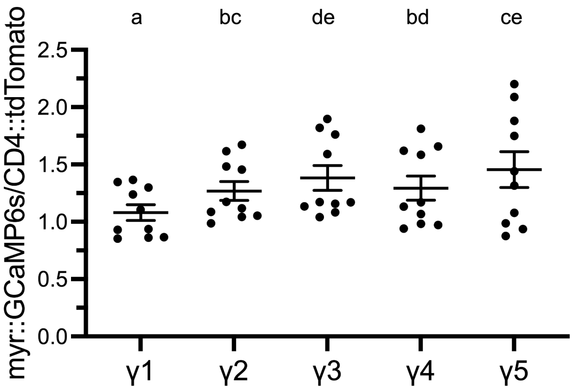

Fig. S8 Basal Ca2+ concentrations in different γ compartments measured by myr::GCaMP6s. The ratio of myr::GCaMP6s to CD4::tdTomato in γ compartments is shown. Experimental procedures and quantification are comparable to that of Fig. 2E. Error bars show S.E.M. Significant differences ($P < 0.05$) are indicated by distinct letters. Repeated measures one-way ANOVA.

We have also measured the GCaMP6s/mCherry fluorescence ratio in fixed brain samples expressing Brp::mCherry::GCaMP6s in KCs and found the ratio is indistinguishable across compartments except for a slightly lower ratio in γ 5 (Fig. R4). Together, these control experiments confirm that the compartmental pattern seen in live animals is dependent on physiological calcium levels.

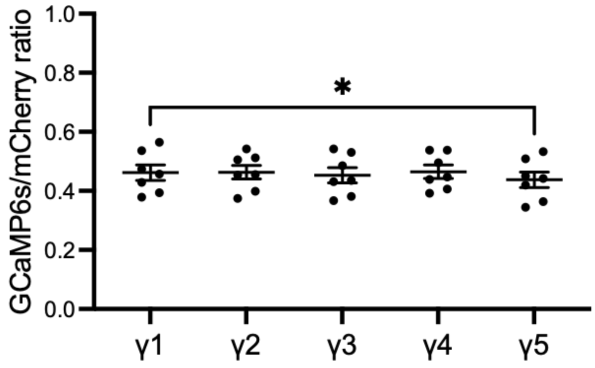

Fig. R4 The ratio of GCaMP6s to mCherry fluorescence in fixed brain samples expressing Brp::mCherry:GCaMP6s in KCs. *$P < 0.05$. Repeated measures one-way ANOVA.

3) The data shown in figure 3A are differentiated by compartment, with gamma1 showing a low level of heterogeneity (while fed). It is not clear in the starvation experiments (Fig. 3B, 4E) which compartment(s) are included in the analysis - all of the gamma compartments? Since the OA/TA innervation is biased toward gamma1, is this quantification focused on the compartment where most of the relevant plasticity may be expected to occur? Did gamma1 show a difference relative to the other compartments?

Quantification of the Brp heterogeneity levels in this study considers all 5 compartments of γ KCs. We apologize for the confusion. In the revision, we clarified the procedures of data processing for quantifying compartmental heterogeneity by including graphical explanation (Fig. 3A) and show all raw data (median intensity of Brp::rGFP clusters for each γ compartment; Fig. S11 and S12). These figures detail how data from all five compartments are processed to generate a single heterogeneity value per brain.

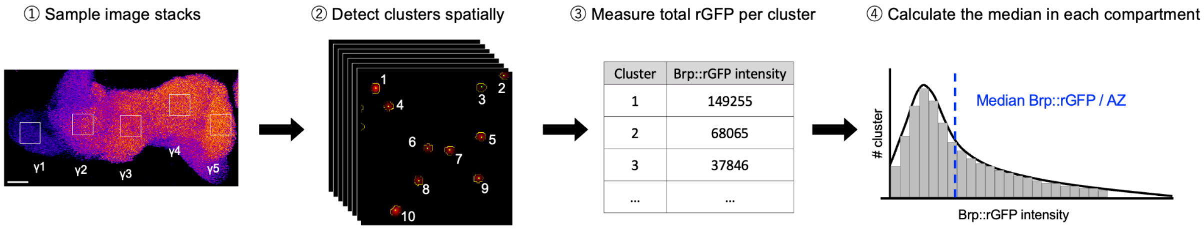

Fig. 3A Graphical summary of the image analysis. 3D volumes were sampled from different γ compartments. Brp::rGFP clusters are then spatially detected using the 3D spot segmentation Fiji plugin. rGFP signal of individual clusters was measured and the median intensity is calculated for each compartment.

Minor typo in title on page 35: "supplementary infroamtion”

We have corrected the typo.
